# Expression of quasi-equivalence and capsid dimorphism in the Hepadnaviridae

**DOI:** 10.1371/journal.pcbi.1007782

**Published:** 2020-04-20

**Authors:** Weimin Wu, Norman R. Watts, Naiqian Cheng, Rick Huang, Alasdair C. Steven, Paul T. Wingfield

**Affiliations:** 1 Laboratory of Structural Biology Research, National Institute of Arthritis and Musculoskeletal and Skin Diseases, National Institutes of Health, Bethesda, Maryland, United States of America; 2 Protein Expression Laboratory, National Institute of Arthritis and Musculoskeletal and Skin Diseases, National Institutes of Health, Bethesda, Maryland, United States of America; Bogazici University, TURKEY

## Abstract

Hepatitis B virus (HBV) is a leading cause of liver disease. The capsid is an essential component of the virion and it is therefore of interest how it assembles and disassembles. The capsid protein is unusual both for its rare fold and that it polymerizes according to two different icosahedral symmetries, causing the polypeptide chain to exist in seven quasi-equivalent environments: A, B, and C in AB and CC dimers in T = 3 capsids, and A, B, C, and D in AB and CD dimers in T = 4 capsids. We have compared the two capsids by cryo-EM at 3.5 Å resolution. To ensure a valid comparison, the two capsids were prepared and imaged under identical conditions. We find that the chains have different conformations and potential energies, with the T = 3 C chain having the lowest. Three of the four quasi-equivalent dimers are asymmetric with respect to conformation and potential energy; however, the T = 3 CC dimer is symmetrical and has the lowest potential energy although its intra-dimer interface has the least free energy of formation. Of all the inter-dimer interfaces, the CB interface has the least area and free energy, in both capsids. From the calculated energies of higher-order groupings of dimers discernible in the lattices we predict early assembly intermediates, and indeed we observe such structures by negative stain EM of *in vitro* assembly reactions. By sequence analysis and computational alanine scanning we identify key residues and motifs involved in capsid assembly. Our results explain several previously reported observations on capsid assembly, disassembly, and dimorphism.

## Author summary

Hepatitis B virus has infected approximately one third of the human population and causes almost 1 million deaths from liver disease annually. The capsid is a defining feature of a virus, distinct from host components, and therefore a target for intervention. Unusually for a virus, Hepatitis B assembles two capsids, with different geometries, from the same dimeric protein. Geometric principles dictate that the subunits in this system occupy seven different environments. From comparing the two capsids by cryo-electron microscopy at high resolution under the exact same conditions we find that the polypeptide chains adopt seven different conformations. We use these structures to calculate potential energies (analogous to elastic deformation or strain) for the individual chains, dimers, and several higher-order groupings discernible in the two lattices. We also calculate the binding energies between chains. We find that some groupings have substantially lower energy and are therefore potentially more stable, allowing us to predict likely intermediates on the two assembly pathways. We also observe such intermediates by electron microscopy of *in vitro* capsid assembly reactions. This is the first structural characterization of the early assembly intermediates of this important human pathogen.

## Introduction

Hepatitis B virus (HBV) constitutes a major health hazard in many parts of the world, particularly in Asia and Africa, killing more people than HIV, malaria, or TB [1]. It is estimated that *ca*. 257 million people are infected with the virus resulting in almost 1 million deaths annually, primarily from complications due to liver cirrhosis and hepatocellular carcinoma [2]. The virion is composed of a structurally distinctive capsid (called core-antigen) containing the genome and polymerase, and surrounded by a lipid envelope with embedded surface-antigen protein. The capsid structure has been extensively studied by electron microscopy, X-ray crystallography, and NMR spectroscopy [3–10]. Capsids are composed of a 183-residue polypeptide, the first 140 residues of which suffice for capsid assembly [11]. The fold of this assembly domain (fold superfamily FSF a.62.1) is unusual [12]. It belongs to the Retrotranscribing-like lineage which has only two members–*Retroviridae* and *Hepadnaviridae*. It is not clear whether this similarity is due to convergent or divergent evolution [12]. The assembly domain is also unusual in that it polymerizes according to two different geometries i.e. T = 3 and T = 4 icosahedral quasi-symmetry. Accordingly, the same polypeptide chain exists in seven quasi-equivalent environments (A, B, and C in T = 3 capsids, and A, B, C, and D in T = 4 capsids). There are also four quasi-equivalent dimers (AB and CC in T = 3 capsids, and AB and CD in T = 4 capsids) [11]. Assembly has been proposed to begin, at least *in vitro*, with the formation of a nucleus, a triangular trimer-of-dimers, and then to proceed by the addition of further dimers [13,14]. Early assembly intermediates, while detected by mass spectrometry [15–17], have not been structurally characterized.

Capsids of both sizes are formed in the cytoplasm during infection, with the majority (*ca*. 95%) being of the T = 4 form [18,19]. Some capsids contain the viral genome but the majority are empty [20,21]. Some filled capsids enter the nucleus, presumably to further the infection [22]. Both filled and empty capsids become enveloped as they are secreted from cells and have been found in the serum of infected patients [23,24]. Some filled but non-enveloped particles are also secreted from cells by a different pathway [25,26]. The filled enveloped particles are deemed to be infectious virions (called “Dane particles”). Roles in infection and pathogenesis have yet to be assigned to the filled-but-nonenveloped, and empty-but-enveloped, capsids [20,25,26]. HBV also codes for e-antigen, a 159-residue protein colinear with the core-antigen assembly domain but having a 10-residue N-terminal propeptide and missing the C-terminal arginine-rich tract of the 183-residue core-antigen. The e-antigen also forms dimers, although in an arrangement different from that of the core-antigen [27]. In a reducing environment, dimeric e-antigen can rearrange into a core-antigen-like dimer and assemble into T = 3 capsids [27,28]. The dimeric form of e-antigen is thought to function as a tolerogen, modulating the immune response to core-antigen [29–32]. The role of the T = 3 e-antigen capsid *in vivo*, if any, remains unknown.

HBV is not unique to man as similar viruses also infect several families of primates, rodents, bats, and birds with the potential for zoonotic transmission to humans, and also from humans to domestic animals such as swine and poultry [33]. The recent identification of an endogenized, 99% complete, Hepadnaviral genome in a zebra finch demonstrated the coexistence of these viruses and their hosts in the Upper Cretaceous, >82 million years ago (Mya) [34]. Subsequent discovery of endogenized Hepadnaviral genomes in a crocodilian, snake, and turtle moved the date of the earliest endogenization event to the Early Mesozoic, >270 Mya. The characteristic compact organization of the Hepadnaviral genome, including the overlapping and nested genes, and the structure of the viral replicase, was found to be highly conserved since that epoch [35,36]. More recently, phylogenetic reconstruction of the Hepadnaviridae and a diverse family of non-enveloped fish viruses lacking the envelope open reading frame, and therefore designated as Nackednaviruses, indicated that these two families of viruses probably diverged in the Silurian, >400 million Mya, before the appearance of tetrapods. The capsids of these viruses are very similar to those of HBV but form with exclusively T = 3 symmetry [37].

Apart from gaining an improved understanding of viral capsid structure, assembly, and disassembly–for instance as a target for assembly-disrupting drugs [5,38–41] and inhibitory antibodies [42]–there is also the prospect of engineering capsids for epitope display and as delivery vehicles [43–46]. These considerations have motivated the determination of a substantial number of structures for HBV capsids. Currently, the Electron Microscopy Data Bank (EMDB) and the Protein Data Bank (PDB) include 51 non-redundant HBV capsid-related structures (33 in the EMDB, 18 in the PDB) (S1 Table). Of these, all but four are T = 4 structures, and of the T = 3 structures, two are from Nackednaviruses reconstructed to low (8.0–9.0 Å) resolution [37], one to intermediate resolution (5.6 Å) [47], and one (at 4.0 Å) is ligand-bound [41]. The highest resolution reported for T = 4 capsids by X-ray crystallography is 3.4 Å [4] and more recently by cryo-EM (3.5 Å) [8]. The highest resolution attained for the T = 3 capsid (4.0 Å) is for the liganded structure [41]. No high-resolution structure for the T = 3 apo form has been reported.

All currently available HBV capsid protein models differ from one another, even ones nominally the same, hindering comparison and interpretation. It is likely that, despite their robust appearance, the inherent flexibility of the capsids, as evidenced by all-atom molecular dynamics simulations, is an important contributor to this variability [48]. In addition to flexibility, several other factors may contribute to model variability. These include sequence differences (both natural and engineered), buffer composition, whether and which ligands are present, method of data acquisition (X-ray or cryo-EM), and for the latter, the instrument used, imaging conditions employed, the numbers of particle images collected, on how a reconstruction is performed and how the models are built.

To better compare the T = 3 and T = 4 capsids, we have attempted to minimize all these experimental variables by: fractionating and then remixing the two morphs in equal numbers; recording data under identical buffer and cryo-EM imaging conditions (i.e. on the same grid); calculating reconstructions in parallel to the same resolution; and building both atomic models in identical fashion. By several criteria, the resulting structures compare favorably with the best available reference models. In this way, it was possible to compare, directly and quantitatively, the conformations of the quasi-equivalent chains both within and between the two morphs. Potential energy calculations show distinct differences between the chains, dimers, and several superficially similar forms of two higher-order subassemblies, both within and between the two morphs, suggesting local strain in the capsid lattices. Free energy calculations show distinct differences between both intra- and inter-dimer interfaces in the two morphs. Sequence comparison of the capsid proteins from Hepadnaviruses from a broad range of host taxa, including ones capable of forming only the T = 3 form, identify features that correlate with morphology. In this paper, we first present our structures in context with existing ones and then discuss how our observations from the above analyses relate to capsid assembly, disassembly, and dimorphism.

## Results

### Reconstruction and modeling of the T = 3 and T = 4 capsids

**Fig 1:**
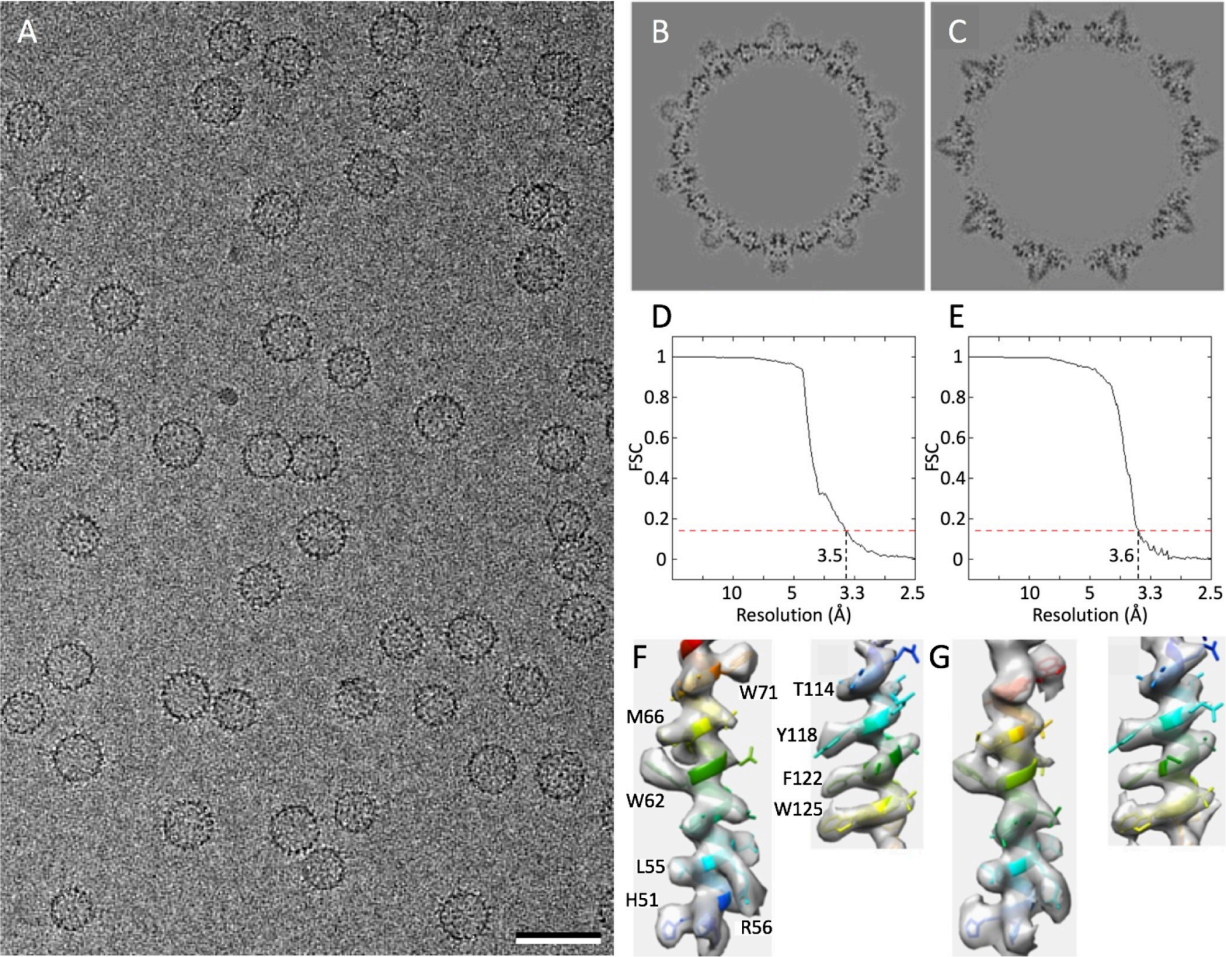
Cryo-EM reconstruction of Hepatitis B virus T = 3 and T = 4 capsids. (A) Field of capsids remixed in equal numbers. (B, C) Central sections through the respective density maps. (D, E) Fourier shell coefficient curves showing that the capsids have been reconstructed to essentially the same resolution (3.5 and 3.6 Å, respectively, at the FSC = 0.143 criterion). (F, G) Fit of sidechains into the density maps of T = 3 (F) and T = 4 (G) capsids at helix 3 (residues 51–71, left) and helix 5 (residues 114–125, right). Bar = 50 nm.

**Fig 2:**
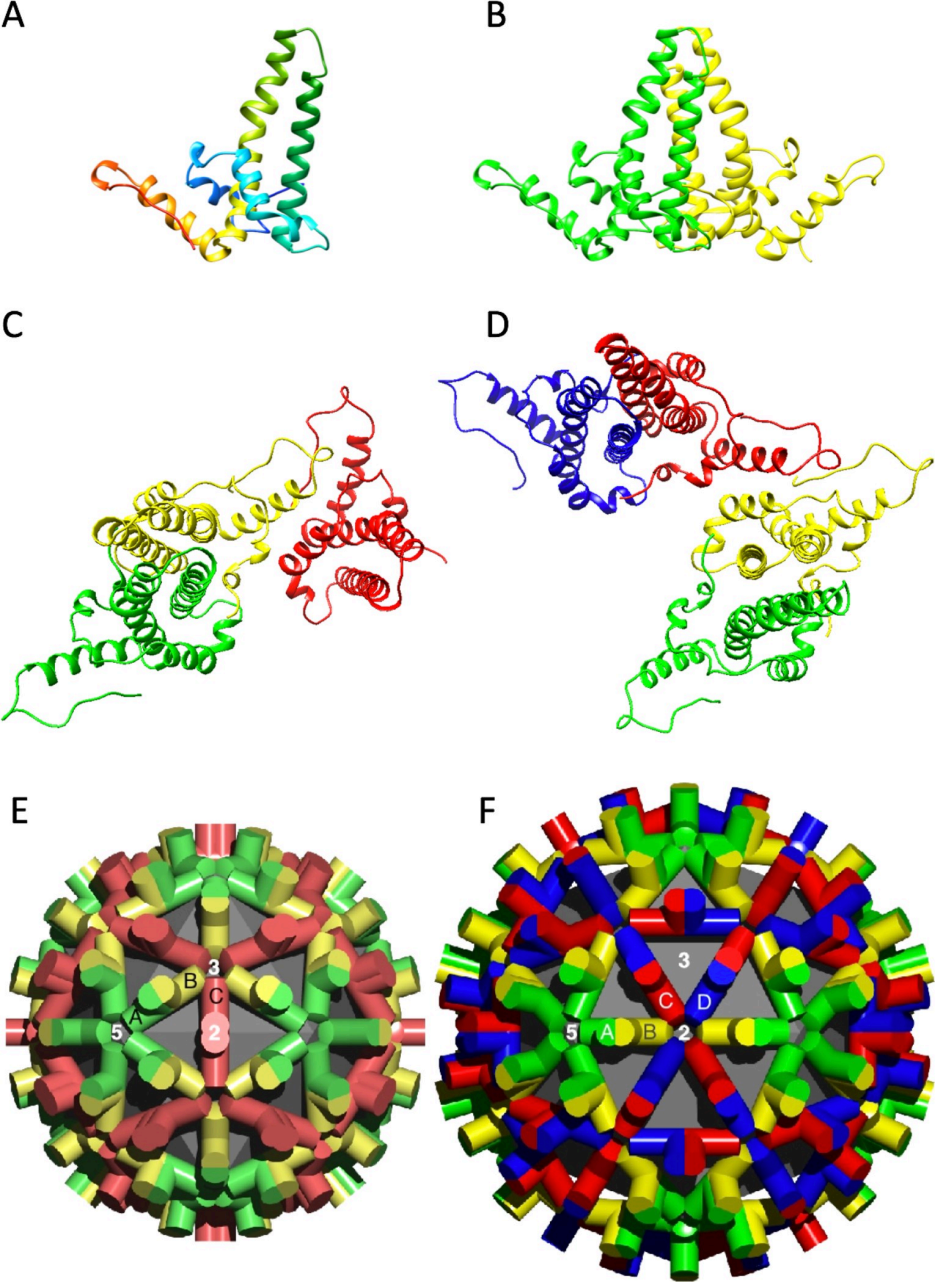
Conformation of core protein and its arrangement in capsids. (A) Ribbon diagram of a single chain colored from N-terminus (blue) to C-terminus (red). Such chains do not exist in isolation. (B) An AB dimer with the A and B chains colored green and yellow, respectively. (C) The A, B, and C chains of T = 3 capsids colored green, yellow, and red, respectively. (D) The A, B, C, and D chains of T = 4 capsids colored green, yellow, red, and blue, respectively. (E, F) Lattices of the T = 3 and T = 4 capsids with the subunits labeled and colored according to the same convention. The chains in (C, D) are shown approximately as they are arranged in the corresponding lattices (E, F). The different shades in the lattices indicate quasi-equivalence.

In a typical *in vitro* reaction with the 149-residue long assembly domain construct (Cp149) the T = 3 and T = 4 capsids are formed in the ratio of *ca*. 5:95, respectively [49], necessitating an increase of the T = 3 fraction to obtain similar numbers of particles in the same field of view. To record images of the two capsid forms in equal numbers, and, more importantly, under identical buffer and imaging conditions to allow direct comparison, the particles were fractionated on sucrose gradients and then remixed in equal proportions (Fig 1A). The T = 3 and T = 4 capsids were reconstructed to essentially the same final resolution– 3.5 and 3.6 Å, respectively (Fig 1B–1E), and atomic models built into the resulting density maps (see Materials and Methods). In most instances it was possible to fit sidechains into the densities with confidence (Fig 1F and 1G). Models of the seven quasi-equivalent chains and their mutual arrangements are shown in Fig 2. Several structures for the T = 4 capsid, both apo and liganded with antivirals, have been reported [3–6,39,50,51], and a 4.0-Å structure for the T = 3 capsid in the ligand-bound form is also available [41]. The structure of the T = 3 capsid described here is the first at high resolution for the apo form.

Our T = 3 and T = 4 structures align well with existing reference models, but in all cases there are numerous local differences, and these differences vary depending on the reference chosen. The quality of the current models was assessed with *MolProbity*, a tool for macromolecular structure validation [52], and compared with ten HBV capsid protein structures in the PDB with similar resolution (Tables 1 and S2). These reference structures were determined by both X-ray crystallography and cryo-EM and they include eight capsids (one T = 3 and seven T = 4) and two non-capsid forms. The reference set further includes four apo structures and six liganded ones (five capsid and one non-capsid). By most measures reported by *Molprobity*, our T = 3 and T = 4 capsid models compare favorably with the reference set. For the all-atom clash score, our T = 3 and T = 4 models are in the 97^th^ and 99^th^ percentile (N = 1784, all resolutions), respectively. In terms of protein geometry, as summarized in the composite *MolProbity* score, our models are in the 96^th^ and 97^th^ percentile (N = 27675, 0 Å – 99 Å), respectively. These results show that the two capsid structures have been determined to the same high quality, and validated comparing the conformations of the same polypeptide chain not only in different symmetry locations within a capsid but also between the two morphs.

**Table 1:** Comparison of the quality of the current structures with several reference structures.

PDB code	1QGT	2G33	2G34	3J2V	3KXS	4G93	5D7Y	5E0I	6BVF	6BVN	This study		
Structure	T = 4	T = 4	T = 4	T = 4	-	T = 4	T = 4	-	T = 4	T = 3	T = 3	T = 4	
*Molprobity* score	Score^1^												Goal^2^
Clash (all atom)^3^	18^th^	16^th^	13^th^	97^th^	30^th^	40^th^	97^th^	98^th^	78^th^	89^th^	97^th^	99^th^	-
Poor rotamers	28.46	8.93	10.16	9.90	6.77	2.0	12.24	2.99	0.00	0.00	0.00	0.00	<0.3
Favored rotamers	53.95	76.19	79.08	82.33	85.25	87.58	77.14	93.08	98.98	99.46	96.76	98.79	>98
Ramachandran outliers	8.19	18.28	25.26	0.70	3.58	11.17	3.02	0.00	0.00	0.00	0.00	0.00	<0.05
Ramachandran favored	66.73	55.17	45.30	95.45	90.21	57.66	88.08	96.46	92.14	95.54	95.25	94.66	>98
*Molprobity* score^4^	7^th^	30^th^	19^th^	97^th^	17^th^	67^th^	85^th^	81^th^	98^th^	100^th^	96^th^	97^th^	-
Cß deviation (>0.025 Å)	0.18	0.35	0.72	0.00	0.25	0.00	0.92	0.94	0.00	0.00	0.00	0.00	0
Bad bonds	0.00	0.00	0.00	0.00	0.03	0.06	0.04	0.04	0.00	0.00	0.00	0.00	0
Bad angles	0.17	0.21	0.28	0.05	0.13	0.00	0.08	0.04	0.02	0.02	0.00	0.00	<0.1

See S2 Table for a more inclusive form of this table.

^1, 2^ All score values are percent (%) except Clash score and *Molprobity* score, which are percentile, as defined below.

^3^ Clash score is the number of serious steric overlaps (>0.4 Å) per 1,000 atoms.

^4^ *Molprobity* score combines clash score, rotamer, and Ramachandran evaluations into a single score, normalized to be on the same scale as X-ray resolution. For both Clash score and *Molprobity* score the values are percentile (100^th^ is best, 0^th^ is worst) relative to a set of comparable structures determined for each calculation (see *Molprobity* server for details).

Colors compare score versus goal; green (best), yellow (intermediate), orange (worst). Adapted from *Molprobity* website.

### Quasi-equivalent core-antigen subunits have different conformations

The basic fold of HBV capsid polypeptide chains, their pairing as dimers, and the arrangement of dimers in T = 3 and T = 4 lattices (Fig 2) have been well established by cryo-EM [3,8], X-ray crystallography [4,5], and NMR [6,9,10]. There have also been indications that the chain conformations, while similar, are not identical [7–9,53]. Of the eight capsid structures given in Table 1, only three (1QGT, 2G33, and 3J2V) are non-liganded and these are all T = 4. We initially chose these three as the closest reference structures. The structure nominally most similar to our T = 4 capsid is 3J2V, both in terms of the methodology used (cryo-EM) and the resolution attained (3.5 Å). For 3J2V, the Cα-RMSD between the chains were previously reported to be: AB (0.868 Å), AC (0.700 Å), and AD (0.632 Å) [8]. In the current T = 4 structure the corresponding Cα-RMSD values are similar: AB (0.792 Å), AC (0.737 Å), and AD (0.603 Å). These numbers illustrate some of the variability that is observed even when the structures compared are nominally the same and are processed in essentially the same way. Other pairs of structures differ even more.

To assess the influence of dimers being in either a capsid or non-capsid environment, all-atom RMSDs were calculated for all pairwise combinations (AB, AC, AD, BC, BD, and CD) of the chains in the current T = 4 structure, the three non-liganded reference T = 4 structures (1QGT, 2G33, and 3J2V), and a non-liganded non-capsid structure (3KXS) (S3 Table). These results further illustrate the variability of these structures. They also suggest that the T = 4 capsids have greater differences between some of their chain pairs, and overall are somewhat similar to one another, whereas the non-capsid structure has fewer differences between its chains and appears distinct from the capsids. One other difference is clear: non-capsid structures achieve much higher resolution than capsid structures (S2 Table).

To assess the influence of ligand binding, we compared our non-liganded T = 3 and T = 4 structures with the only available pair of liganded T = 3 and T = 4 capsids (6BVN and 6BVF). Comparison of the all-atom RMSD of the liganded and non-liganded capsids (Table 2) showed that whereas the A, B, and C chains in T = 3 and T = 4 capsids were similar to each other, respectively, in the free state, this was not the case in the liganded state where the C chains were different from one another and T = 4 D chains were different from A chains. This change in the C and D chains may reflect the fact that the ligand binds at both ends of CC dimers in T = 3 capsids and CD dimers in T = 4 capsids [41]. These results illustrate in just one case how ligands can perturb the structure even when only assessed by overall RMSD.

**Table 2:** All-atom RMSD (Å) of chain pairs in apo and liganded T = 3 and T = 4 capsids^1^.

**Apo T = 3 versus T = 4 (This study)**							
	T = 3			T = 4			
Chain	A	B	C	A	B	C	D
**T = 3 A**	0.00	0.85	1.09	**0.57**	0.93	1.06	0.69
T = 3 B		0.00	1.27	0.95	**0.74**	1.29	0.98
**T = 3 C**			0.00	1.13	1.38	**0.65**	1.11
T = 4 A				0.00	0.88	1.05	**0.58**
**T = 4 B**					0.00	1.33	0.90
T = 4 C						0.00	1.05
**T = 4 D**							0.00
**Liganded T = 3 versus T = 4 (6BVN / 6BVF)**							
	T = 3			T = 4			
Chain	A	B	C	A	B	C	D
**T = 3 A**	0.00	0.90	1.34	**1.07**	0.91	1.58	1.05
T = 3 B		0.00	1.59	1.15	**0.81**	1.71	1.24
**T = 3 C**			0.00	1.37	1.39	1.63	1.07
T = 4 A				0.00	1.20	1.71	1.21
**T = 4 B**					0.00	1.56	1.25
T = 4 C						0.00	1.38
**T = 4 D**							0.00

^1^ For the apo T = 3 and T = 4 capsids described in this study, the respective A, B, and C chains have the most similar conformations, and the T = 4 A and D chains are similar to each other. For the liganded T = 3 and T = 4 capsids (6BVN / 6BVF), the respective A and B chains have the most similar conformations, but the C and D chains (where the HAP-TAMRA molecule is bound) have altered conformations. Overall similar conformations (low RMSD values) are shown bold.

To visualize the conformational differences between quasi-equivalent chains in the current T = 3 and T = 4 structures, the ribbon diagrams were color-coded according to Cα-RMSD relative to the T = 3 C chain (S1 Fig). The hydrophobic core region, corresponding to the bases of helices 3, 4, and 5, shows the least difference and the C-terminal region the greatest. In agreement with the average RMSD values, the T = 3 A, B, and C chains appear similar to the T = 4 A, B, and C chains, respectively. The T = 3 C chain and T = 4 C chain also appear similar to one another, as do the T = 4 A and D chains. Differences between the chains also occur in the N-terminal region between helices 1 and 2. Surprisingly, little difference in RMSD was observed in the regions between helices 3 and 4, parts of the structure that are flexible. This observation is also made with a dynamic analysis (see section on dynamic properties). Taken together, these results show that the greatest conformational differences between chains are located in the C-terminal domains, and that while CC dimers are symmetric, AB and CD dimers are asymmetric with respect to the conformations of their constituent chains.

**Fig 3:**
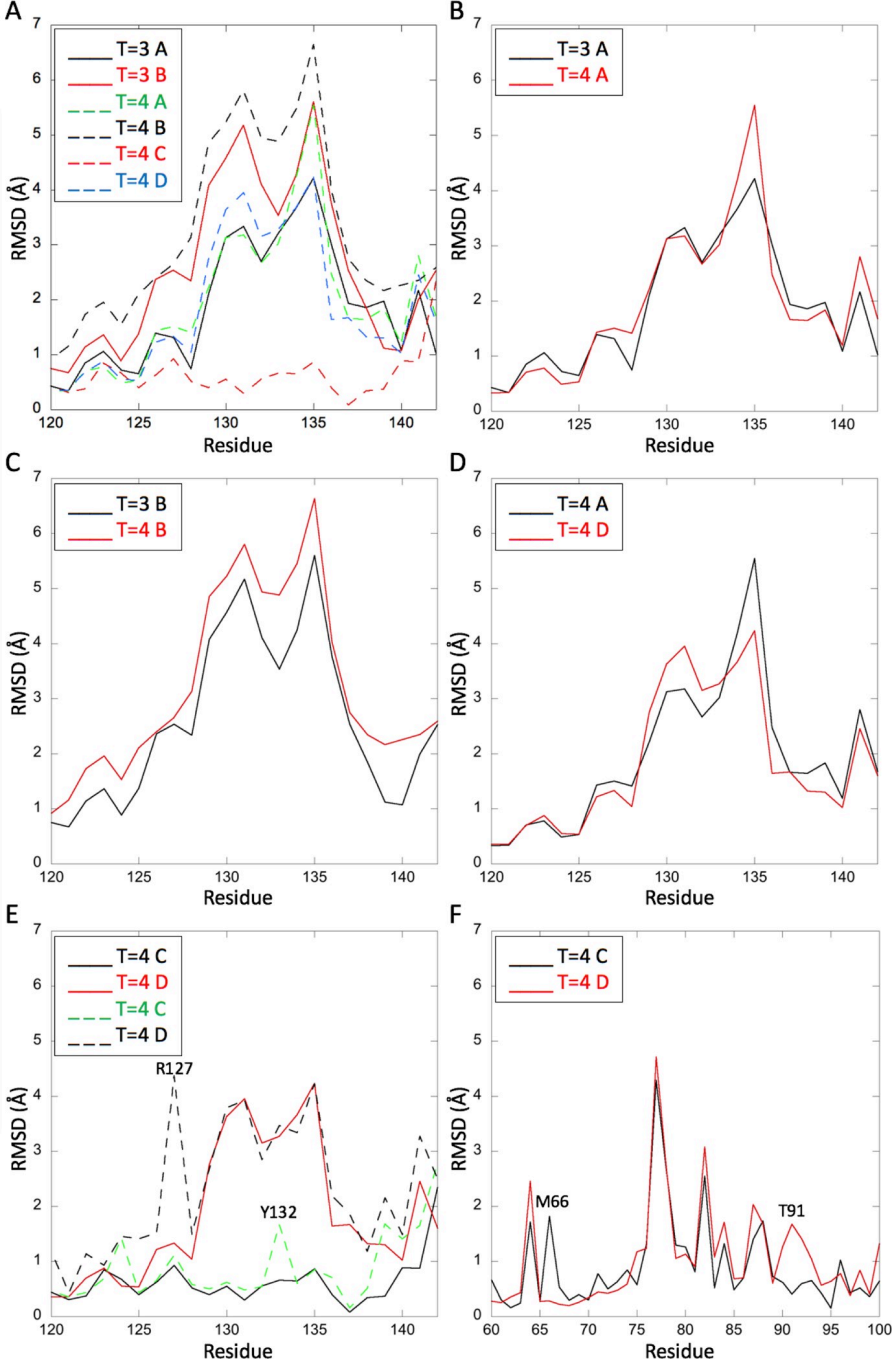
RMSD plots of the six other chains in T = 3 and T = 4 capsids aligned to the T = 3 C chain. (A) Cα-RMSD plots for all six chains, between residues 120–142, showing that the C chains are similar to each other and distinct from the others. (B-D) Cα-RMSD plots for the chain pairs indicated in the legends, between residues 120–142, showing their mutual similarity. (E) Cα (solid line) and all-atom (dashed line) RMSD plots for the T = 4 C and D chains, between residues 120–142, showing differences at residues R127 in the T = 4 D chain and Y132 in the T = 4 C chain. (F) All-atom RMSD plots for the T = 4 C and D chains, between residues 60 and 100, showing differences at residues M66 in the T = 4 C chain and T91 in the T = 4 D chain.

Comparison of the Cα-RMSD between the T = 3 C chain and the six other chains in the current T = 3 and T = 4 capsids shows qualitatively that the greatest differences lie in the C-terminal regions (S1 Fig). Cα-RMSD plots of the region between residues 120 and 142 show quantitatively that the T = 3 and T = 4 C chains have a conformation different from the other five chains, and that the two A chains, the two B chains, and the T = 4 A and D chains are similar, respectively (Fig 3). Furthermore, comparison of Cα- and all-atom RMSD in this region highlights the differences between the T = 4 C and D chains at residues R127 and Y132 (Fig 3E). Both of these residues play an important role at the inter-dimer interface as their mutation to Alanine blocks capsid assembly [54,55]. These residues are also identified as key by mutational alanine scanning (see section on free energies).

The conformations of the T = 4 C and D chains relative to the T = 3 C chain also differ distinctly in their N-terminal regions at residues M66 and T91, respectively (Fig 3F). It is worth noting that residue M66 is located in helix 3 adjacent to the hinge region between helix 4a and helix 4b, which is bounded by residues T91 and K96. K96 is highly conserved across genotypes A, B, C, and D, is a key ubiquitination site involved in interactions with the surface antigen, and it appears to be involved in conformational changes between RNA- and DNA-containing capsids and therefore capsid maturation and nucleocytoplasmic trafficking [56–58]. Notably, this region of the sequence is also very different in the core antigen of the non-enveloped fish viruses (see section on phylogeny).

### Quasi-equivalent core-antigen subunits have different dynamic properties

To more specifically define the conformational and potentially also the dynamic differences between chains we employed *DynDom* [59]. *DynDom* is a tool used to compare two conformations of the same protein for the quantitative assignment of hinge and shear movements between domains. It also provides animation to enable the user to visualize the domain motions, including those that may not correspond to an obvious cluster. *DynDom* has been used to characterize domains and hinges detected in core-antigen by solid-state NMR [9]. Here, *DynDom* was used to analyze the pairwise chain combinations in the current T = 3 and T = 4 structures, in three apo reference structures (1QGT, 2G33, and 3J2V), in three liganded structures (4G93, 6BVF, and 6BVN), and in an apo non-capsid structure (3KXS). The analysis was also extended to (hypothetical) pairwise combinations between the chains in the apo T = 3 and T = 4 structures described here and the chains in the liganded T = 3 and T = 4 structures 6BVN and 6BVF (S4 Table).

**Fig 4:**
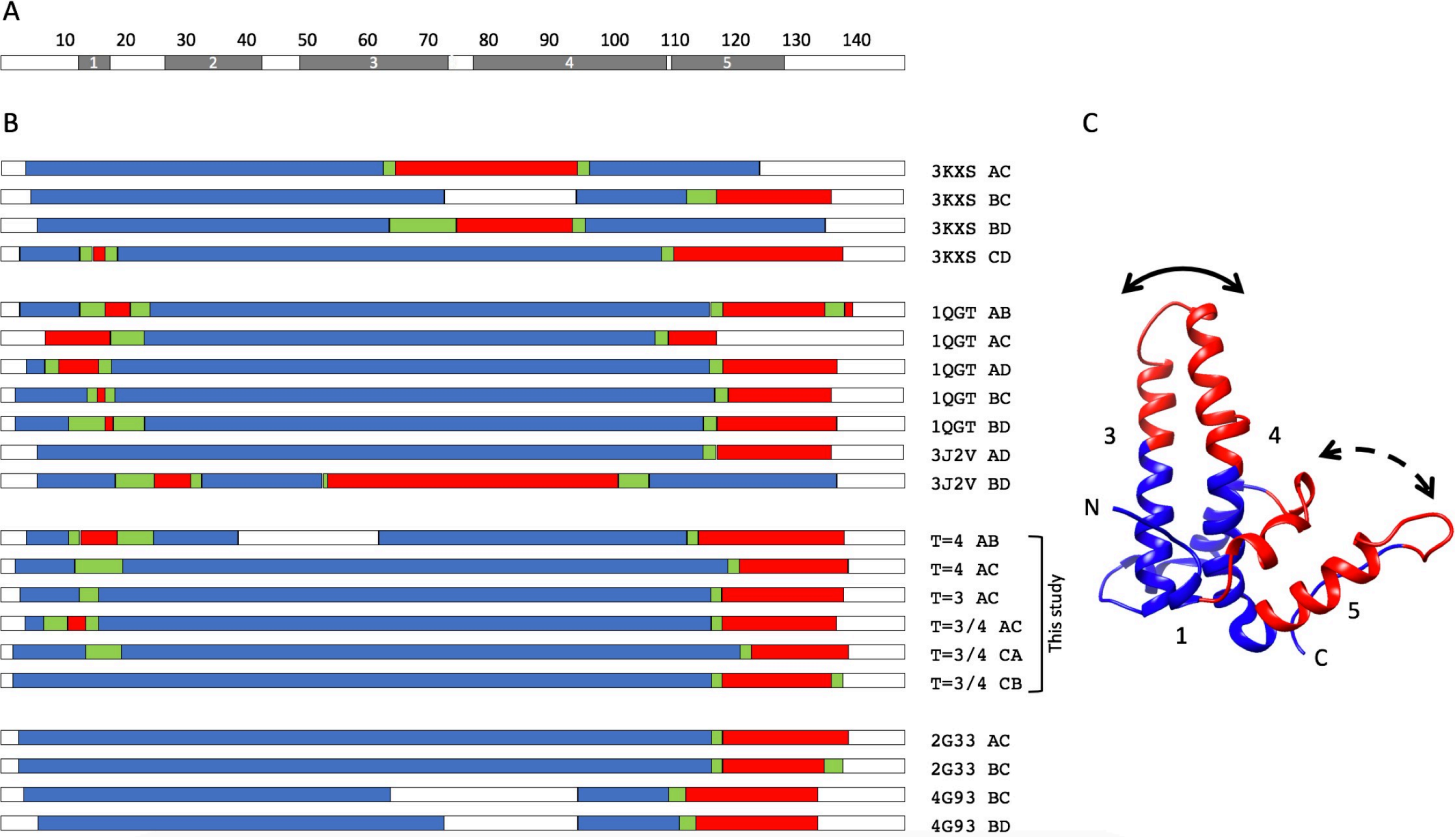
Dynamic domain analysis with *DynDom*. (A) Domain structure of HBcAg, residues 1–149, with helices shown in Grey. (B) Domain assignment by *DynDom*. Blue and Red indicate regions classed as domains, Green indicates residues classed as hinges, Clear regions are undefined. All structures are capsids except 3KXS, all are apo except 2G33 and 4G93, and all are T = 4 except where noted. T = 3/4 denotes comparison of chains *between* the T = 3 and T = 4 capsids described here, in the order given. Of the 72 chain combinations tested (S4 Table), only these 21 were classed as “dynamic” by *DynDom*. (C) Ribbon diagram of a monomer colored as an approximate composite of the domains shown in (B). In *DynDom* animations of chain pairs from soluble dimers the apical region of helices 3 and 4 generally moves relative to the rest of the molecule (solid arrow), whereas, in chain pairs from capsids, helix 5 and the region between helices 1 and 2 move in concert with one another (dashed arrow). This is reminiscent of the “chassis” and “fulcrum” model proposed previously. In soluble dimers (3KXS) and liganded structures (2G33 and 4G93) the “fulcrum” region is generally not detected. Residues beyond 140 are never classed as being in a domain, similar to the observation that constructs truncated before this residue cannot assemble *in vitro*.

Several patterns were observed. The apo capsid structures have two domains, the first involving helices 3 and 4, and the second involving helix 5 plus the residues connecting helices 1 and 2 (Fig 4A and 4B). In animations, the two components of the second domain always move in concert, despite not being an obvious domain upon visual inspection (Fig 4C). This is reminiscent of the core-antigen dimer motions about a “fulcrum” described previously [7]. Unlike the apo capsid structures, the apo non-capsid structure (3KXS) showed a different domain arrangement wherein the region involving helices 3 and 4 (corresponding to part of the four-helix bundle in the context of a dimer, and the apical domain of the spike in the context of a capsid) was classed as a domain distinct from the rest of the structure (Fig 4B). This again shows that dimers in capsids are in a different environment from that in non-capsid crystals. Another observation was that classification of chain pairs as dynamic, or not, varied between nominally similar structures, i.e. 1QGT, 2G33, 3J2V and the T = 4 structure described here. When classed as dynamic, the domain limits, hinge angles, and domain translations in these structures varied. These observations once again point to the conformational variability between structures. Another clear distinction was observed between apo and liganded capsids in the following sense. In a comparison of the 21 possible pairwise combinations of the chains, including 12 chain pairs from both T = 3 and T = 4 capsids (i.e. hypothetical *inter*-capsid pairs), none of the six pairs classed as dynamic in the non-liganded T = 3 and T = 4 capsids described here were classed this way in the liganded T = 3 and T = 4 capsids (6BVN and 6BVF) described previously (S4 Table). This result is similar to that obtained with the all-atom RMSD analysis described above (Table 2), namely, that the binding of ligands broadly disturbs the conformational differences between chains. The *DynDom* analysis illustrates just some of the differences in conformation that are observed both within and between different forms of the core-antigen (e.g. capsid *vs*. non-capsid, T = 3 *versus* T = 4, liganded *vs*. non-liganded), some of which have been modeled in all-atom molecular dynamics simulations of capsids [48].

### Quasi-equivalent core-antigen subunits have different potential energies

The observation above that the quasi-equivalent chains have different conformations raised the question of whether and how much these chains differ in potential energy, i.e. how strained the different chains are. The Molecular Modeling Toolkit (*MMTK*) is an open source program library for molecular simulations and includes energy minimization, typically used to refine structures in X-ray crystallography [60]. Here, *MMTK* energy minimization (implemented as a tool in *UCSF Chimera*) was used to analyze the current T = 3 and T = 4 capsids as well as the ten reference structures in Table 1. The ligands were omitted from the analysis; i.e. only the polypeptide chains were used for the calculations.

It is generally accepted that the HBV capsid protein is an obligate dimer [11]. Consequently, isolated individual chains are hypothetical entities. To examine the potential energies of isolated chains, we first surveyed three reference structures. These included two non-capsid complexes (3KXS and 5E0I) and an apo T = 4 capsid (1QGT). All three are X-ray crystal structures with resolutions of 2.3 Å, 1.9 Å and 3.3 Å, respectively (S2 Table). There is no apo T = 3 capsid X-ray crystal structure available for comparison.

**Fig 5:**
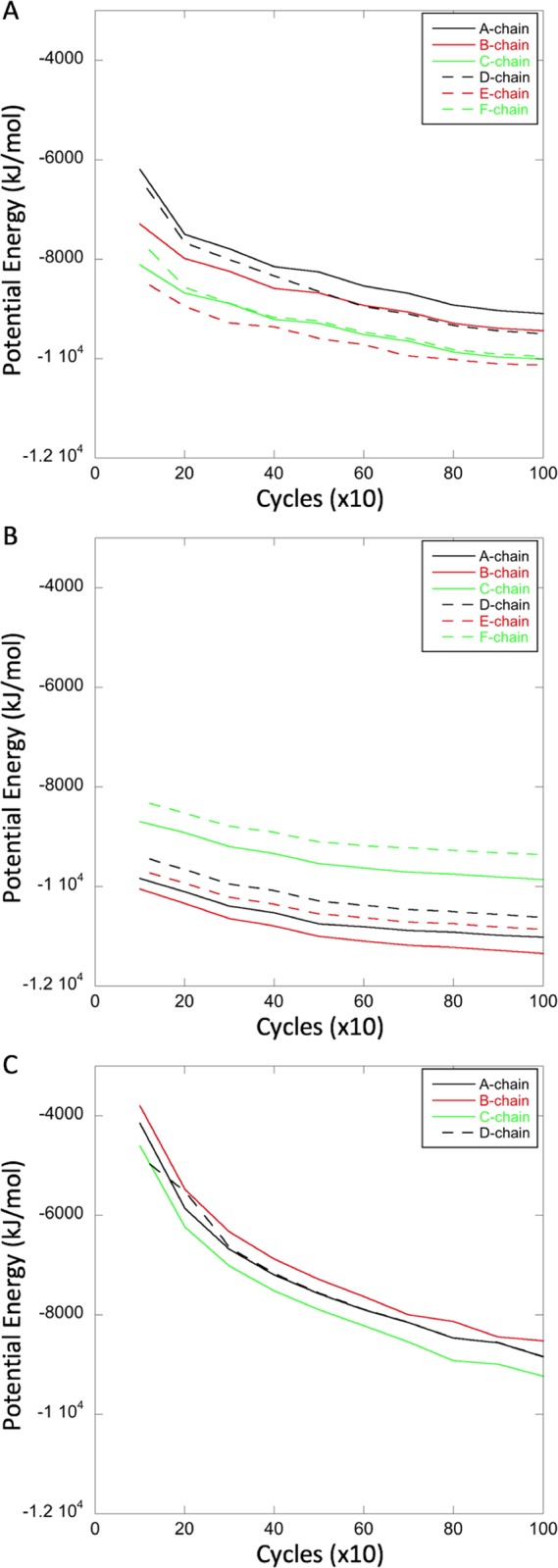
Comparison of potential energy minimization curves of hypothetical monomer chains from non-capsid and capsid dimers. Compared are apo 3KXS (A) and liganded 5E0I (B) non-capsid chains with the apo T = 4 1QGT capsid chains (C). All are X-ray crystal structures of comparable resolution. There is no apo T = 3 capsid X-ray crystal structure available for comparison. The non-capsid profiles (A and B) are similar to each other, and distinct from the capsid profiles (C), showing both lower energy and less decrease with progressive minimization.

For energy minimization, the individual chains were extracted from the structures and minimized in isolation (see Materials and Methods). The potential energy values were calculated and averaged over 10 cycles for a total of 1,000 cycles (Fig 5). The initial potential energy value, i.e. prior to the first average, varied widely and was therefore disregarded. Conjugate gradient minimization was also performed, but, as this resulted in only a minimal change in the values eventualy obtained, the result was not included in the final analysis. The energy minimization trajectories were considered, rather than just a single average value, as these better represent the relationships between the structures. Compared to the chains in non-capsid complexes, the chains in the capsid are at higher potential energy and minimize along a steeper slope, indicating that they are in a more strained conformation (Fig 5).

Energy minimization profiles were then calculated in the same way for the AB and CC dimers extracted from T = 3 capsids, the AB and CD dimers extracted from T = 4 capsids, and the AB, CD, and EF dimers extracted from the non-capsid complexes 3KXS and 5E0I, with the clear understanding that the dimers in the latter two complexes, though in two instances named in the same way as in capsids, are not in a capsid-like environment. For the one apo and liganded T = 4 capsid pair (2G33 and 2G34) the calculation for the apo structure failed to go to completion, precluding a direct comparison for such a pair.

**Fig 6:**
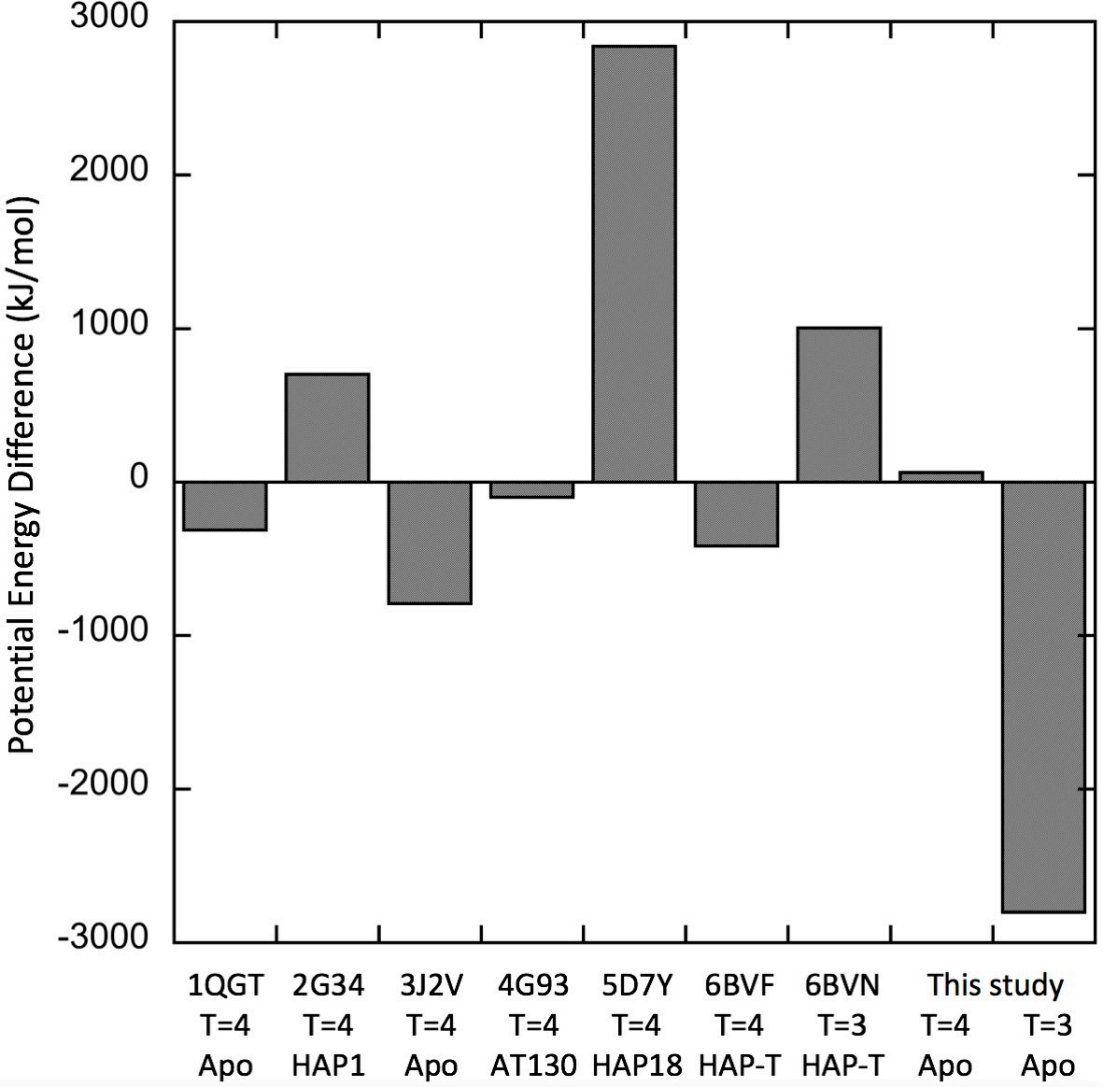
Potential energy difference between AB and CC dimers in T = 3 capsids, and between AB and CD dimers in T = 4 capsids. The subtraction is CC-AB for T = 3 dimers, and CD-AB for T = 4 dimers. The C chain containing dimers CC and CD have a lower potential energy than their corresponding AB dimers, and the T = 3 CC dimer has a particularly low potential energy. Ligands can disturb these relationships.

All the profiles had the same general shape, and while the potential energies of the dimers in nominally similar structures (e.g. 1QGT vs 3J2V) were sometimes different, within a given structure (e.g. 1QGT) they were similar. In fact, within most of the reference T = 4 capsid structures, both apo (1QGT, 3J2V) and liganded (2G34, 4G93, and 6BVF) the AB and CD dimer profiles were similar. However, in some structures the potential energies of the AB and CC dimers (in T = 3 capsids) or AB and CD dimers (in T = 4 capsids) relative to one another were very different or even reversed (e.g. 5D7Y and the T = 3 structure described here). A summary is given in Fig 6. Shown is the difference in potential energy between AB and CC dimers (in T = 3 capsids), and between AB and CD dimers (in T = 4 capsids). In general, in apo structures (1QGT, 3J2V, and the T = 3 capsid structure described here), CC and CD dimers have a *lower* potential energy than their corresponding AB dimers. This is also true in T = 4 capsids treated with the assembly accelerator AT130 which does not distort capsid assembly [39]. By contrast, in three out of four structures (2G34, 5D7Y, and 6BVN) the binding of heteroaryldihydropyrimidine (HAP) allosteric modulators known to misdirect capsid assembly caused the CD dimers to have potential energies *higher* than those of the corresponding AB dimers. HAP18 in particular raised the potential energy of the CD dimer relative to that of the corresponding AB dimer. HAP18 has been shown to affect the tertiary structure of the CD dimer [61]. Hap18 also has a volume (967.1 Å^3^) greater than that of AT130 (817.6 Å^3^) or HAP1 (528.1 Å^3^), suggesting a greater distortion of the structure due to overfilling of the hydrophobic pocket. These results indicate that the C chain-containing dimers CC and CD have a lower potential energy than AB dimers. The CC dimer, having two such chains, has a particularly low potential energy. Taken together, these results showed that while CC dimers are symmetric, AB and CD dimers asymmetric with respect to their potential energy, and that some ligands disturb these relationships.

**Fig 7:**
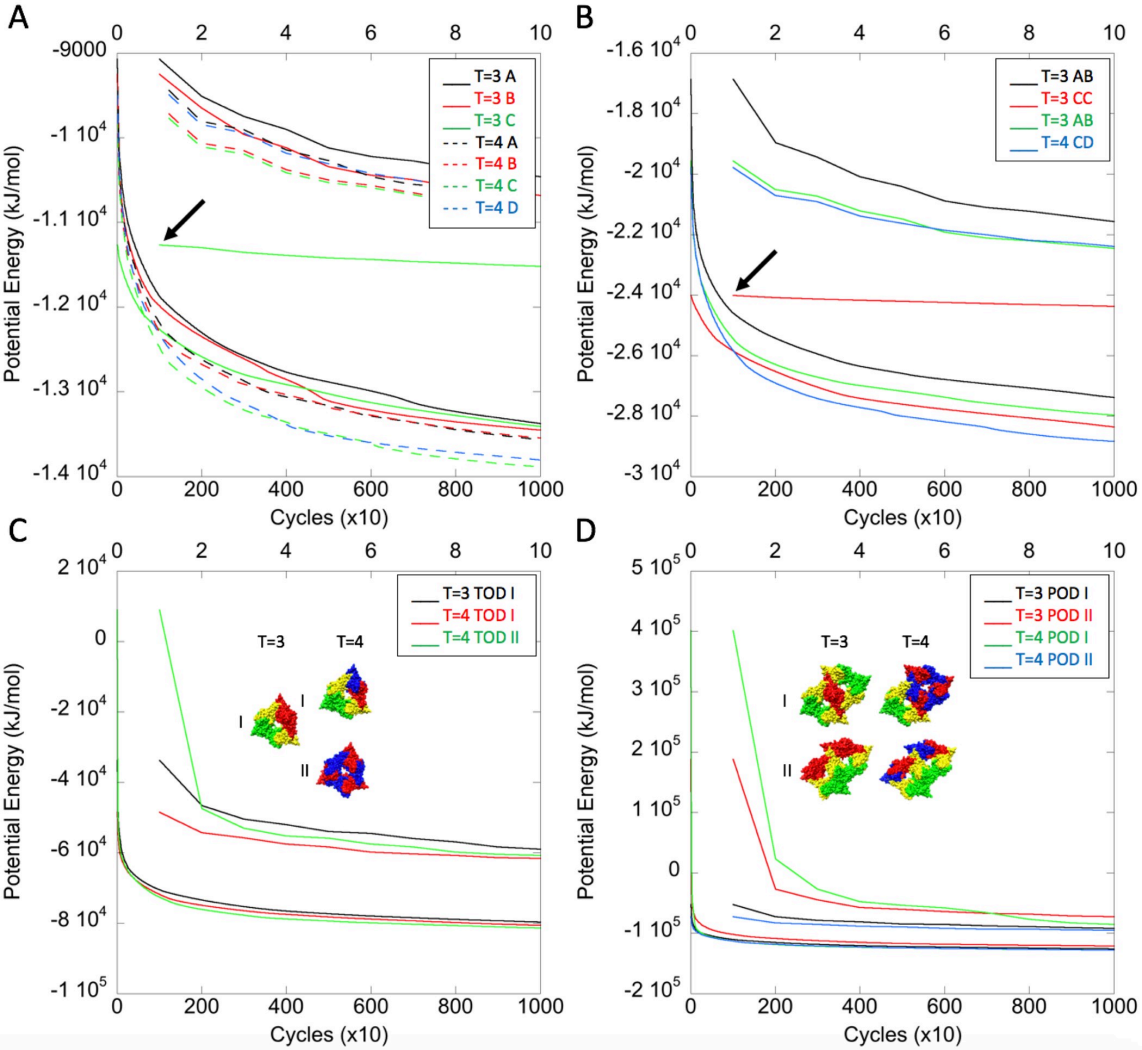
Potential energy minimization of T = 3 and T = 4 capsid substructures. (A) Monomers, (B) Dimers, (C) Trimers-of-dimers, and (D) Pentamers-of-dimers. In all panels, the lower curves show energy minimization for 10,000 cycles and the upper curves show the first 1,000 cycles on an expanded scale (upper X-axis). In each panel, line styles and colors pertain only to that panel. Arrows indicate the curves for the particularly low-energy T = 3 C monomers and T = 3 CC dimers in (A) and (B) as plotted on the expanded X-axis. Models of the Type I and Type II trimers-of-dimers and Type I and Type II pentamers-of-dimers are shown inset in (C) and (D), respectively. For the locations of the structures shown in (C) and (D) in the context of the capsids see Fig 2.

Following these preliminary surveys of a broad range of reference structures, to put the present work in context, we then performed a more extensive analysis focused on our T = 3 and T = 4 capsids. Potential energy minimization profiles were calculated for the 7 monomers, 4 dimers, 3 trimers-of-dimers, and 4 pentamers-of-dimers in these two capsids. The structures were extracted from the capsids and profiles were calculated in the same way as above but this time for 10,000 cycles of minimization at which point the last potential energy decrease for all structures was < 0.04% (Fig 7). The potential energy ranges for the corresponding components (i.e. chain vs chain, dimer vs dimer, etc.) are similar to one another despite originating from two different capsids–validating the experimental approach of recording data under identical buffer and imaging conditions.

**Monomers** (Fig 7A). Similar to the reference structures above (Fig 5), the profiles of the monomers are all monotonically decreasing, but they have different trajectories, occasionally even crossing each other. The T = 3 A chain has the highest potential energy throughout the minimization process and the T = 3 C chain has the lowest with an initial potential energy 24% lower than the T = 3 A chain. By comparison, in eight other capsids from the PDB, chosen to include other folds (Picornavirus, HK97, and BTV), other T-numbers (T = 1, 3, 4, and 7), and monomeric and dimeric subunits, but all unliganded and with similar resolution (3.3–3.6 Å), the maximum difference in potential energy between the corresponding chains was only 7.6%. These results show that C chains, and in particular the C chains in T = 3 capsids, are in a lower energy conformation than all the others in the HBV capsids.

**Dimers** (Fig 7B). The T = 3 AB dimer has the highest potential energy throughout the minimization process. The T = 3 CC dimer initially has a potential energy 42% lower than the T = 3 AB dimer and remains the lowest with continued minimization until replaced as such by the T = 4 CD dimer. These results show that C chain-containing dimers, and in particular the CC dimers in T = 3 capsids, are in a lower energy conformation than all the others.

**Trimers-of-dimers** (Fig 7C). The assembly of capsids has been proposed to begin with the formation of a nucleus, a triangular trimer-of-dimers [13] and we have observed structures suggestive of trimers-of-dimers by EM (see section on negative stain electron microscopy). Three such groupings can be identified in capsid lattices (Fig 2). The three types of trimers-of-dimers are initially quite dissimilar in potential energy, with the T = 4 Type II trimer-of-dimers having a positive energy and the other two negative energy. However, over the remainder of the minimization process the energies of the complexes are more similar. These results show that the potential energies of superficially similar complexes can be quite different, and in some instances even positive.

**Pentamers-of-dimers** (Fig 7D). Similar to trimers-of-dimers, four such groupings can be identified in the capsid lattices (Fig 2) and we observed structures suggestive of pentamers-of-dimers by EM (see section on negative stain electron microscopy). They may form by the addition of two more dimers to a nucleus. Early in the minimization process there are substantial differences between the four types of pentamers-of-dimers: the T = 3 Type I has negative energy while T = 3 Type II has positive energy; conversely, the T = 4 Type I has positive energy while T = 4 Type II has negative energy. Later in the minimization process the four complexes differ little in energy. These results show that, as with trimers-of-dimers, the potential energies of superficially similar complexes can be quite different and that for some the values can be highly positive, suggesting that they are unlikely to form as such.

Comparison of the potential energies of the dimers, trimers-of-dimers, and pentamers-of-dimers with the total energies of the minimized chains that constitute them showed that the relationships were not simply additive, reflecting both intra-dimer and inter-dimer interactions between the chains (see section on free energies). In the case of dimers, the CC dimer gained the most negative energy upon formation, and in the case of trimers-of-dimers, the T = 3 Type I form gained the most negative energy. The T = 4 Type II form has the greatest number of chains in the C conformation (i.e. 3), yet it has a high energy both early and late in the minimization process. Prior to minimization, all four pentamers-of-dimers have energies higher than expected (i.e. relative to the total potential energy of their constituent chains), in the case of the T = 4 Type I and T = 3 Type II complexes these are much higher; following minimization, all four pentamers-of-dimers have energies just slightly lower than expected.

Taken together, the results above show that the polypeptide chains, dimers, and the higher-order complexes likely to form early in capsid assembly, have surprisingly different potential energies. For the higher-order complexes these are not a simple sum of the energies of their constituent chains. These energy differences likely reflect that core-antigen both has two domains that can move relative to one another, and that it is dimeric. As detailed in the Discussion, these differences can explain several previously reported experimental observations of capsid assembly.

### Quasi-equivalent core-antigen subunits have different free energies of association

Quasi-equivalence in viral capsids pertains to both the constituent polypeptide chains and the interfaces between them. In principle, all subunits could have identical conformations and any accommodation would occur at the interfaces. Alternatively, all accommodation could occur in the conformations of the subunits. In actuality, quasi-equivalence likely exists between these two extremes. Above, we demonstrated that the seven quasi-equivalent chains in the T = 3 and T = 4 capsids have distinct conformations and therefore different potential energies. We next calculated the free energies of the interfaces.

We examined the four *inter*-dimer interfaces in T = 4 capsids in five reference structures (1QGT, 2G33, 2G34, 2QIJ, and 4G93) using *PDBePISA*, a tool for examining macromolecular interfaces. The averaged values for several key metrics are summarized in Table 3. These results showed that in T = 4 capsids three of the four interfaces (AA, BD, and DC) are similar to one another but that the CB interface is different, involving fewer residues, a smaller area, and a lower free energy of association. The burial of V124, mutations of which were previously shown to affect the proportion of T = 3 to T = 4 capsids formed during *in vitro* assembly [62], was also found to be less at the CB interface. The same analysis was then performed on the T = 4 capsid described here, with similar results (Table 3). The CB interface involved *ca*. 25% less area, 18% lower free energy, and 42% less burial of V124. The analysis was then extended to the T = 3 capsid (Table 4). Here again, the CB interface was smaller, having *ca*. 20% less area, 19% lower free energy, and 30% less burial of V124. These results also showed that the chains forming this interface are further apart from each other than are chains at the other interfaces, in both T = 3 and T = 4 capsids. A visual impression of the seven interfaces was therefore obtained by rendering them as a surface 3 Å from each chain using the Fast Atomic Density Evaluation (*FADE*) algorithm [63] on the *KFC2* server (S2 Fig). Displayed in this manner, the T = 3 and T = 4 CB interfaces are less continuous and smaller, with volumes 38% and 28% smaller than the average of the others in their respective structures. These results show that quasi-equivalence in HBV capsids involves differences not only in the conformations of the individual polypeptide chains, as shown above, but also in the inter-dimer interfaces between them.

**Table 3:** *PISA* inter-dimer interface analysis of T = 4 capsids^1^.

Structure	Property	AA	BD	CB	DC
**Reference**	N_RES_	46.6	49.4	38.8	38.2
	N_HB_	3.8	4.4	2.8	1.8
	N_SB_	0.6	0.2	3.4	1.0
	Area (Å^2^)	729.2	730.9	562.16	537.8
	ΔG (kCal/mol)	-12.08	-11.96	-7.92	-8.06
	V124 BSA (Å^2^)	63	72	33	46
**This study**	N_RES_	48	53	37	49
	N_HB_	2	1	1	0
	N_SB_	6	1	4	0
	Area (Å^2^)	870.3	870.4	646.6	802.5
	ΔG (kCal/mol)	-14.1	-12.4	-10.3	-11.0
	V124 BSA (Å^2^)	65.3	61.3	36.4	63.2

^1^ Values in the upper half of the table are averages calculated from five reference structures: 1QGT, 2G33, 2G34, 2QIJ and 4G93. Values in the lower half of the table are from the T = 4 capsid described in this study. Here and throughout, interfaces are named with the first given chain fitting into the hydrophobic pocket of the second chain. Viewed from outside the lattice, the interfaces are named clockwise (Fig 2). N_RES_, N_HB_, and N_SB_ are the numbers of residues, hydrogen bonds, and salt bridges, respectively. Free energies are derived from buried surface area. V124 BSA is the buried surface area of V124, a residue observed to be distinctly less buried during preliminary analysis of the reference structures, and also reported to change the T = 3:T = 4 ratio when mutated. Note that the CB interface has the lowest area and free energy values.

**Table 4:** *PISA* inter-dimer interface analysis of T = 3 capsids^1^.

Structure	Property	AA	BC	CB
**This study**	N_RES_	50	50	42
	N_HB_	1	0	0
	N_SB_	6	1	1
	Area (Å^2^)	819.3	842.4	661.4
	ΔG (kCal/mol)	-14.2	-13.0	-11.0
	V124 BSA (Å^2^)	67.9	67.6	47.6

^1^ Only values from the T = 3 capsid described in this study are given as there are no apo T = 3 reference structures available. See footnote to Table 3 for other details. Note that, as in T = 4 capsids, the CB interface has the lowest area and free energy values.

Residues potentially important for binding at the different inter-dimer interfaces were then identified by computational alanine scanning with *KFC2* [64]. *KFC2* employs machine learning to identify residues, or hot spots, where mutation of these to alanine is associated with a change in binding energy (ΔΔG) greater than 2 kCal/mol. Hot spots identified with high confidence are also indicated. In the T = 4 capsid, several residues were identified as hot spots with high confidence (S5 Table). In particular, F23 and F122 were identified with high confidence in all four interfaces, and when these residues were mutated to Alanine and the proteins expressed in *E*. *coli*, capsid expression was either largely (F122A) or completely (F23A) inhibited. Y132, which when mutated to Alanine blocks capsid assembly [54], was also identified in all four interfaces, but with high confidence only in the AA and BD interfaces. In contrast, R127, which when mutated to Alanine enhances the solubility of capsid dimers [55], was identified with high confidence in the AA, BD, and DC interfaces but it was not identified as important at the CB interface. V124, mutations of which influence the dimorphic ratio, was identified as a hot spot in the AA, BD, and DC interfaces but was not identified at the CB interface. The same analysis performed on the three interfaces in the T = 3 capsid again identified F23, F122, and with lower confidence Y132, in all three interfaces (S6 Table). As with the T = 4 CB interface, V124 and R127 were not identified as important in the T = 3 CB interface. Taken together, these results show that the interaction at the CB interface is weaker than at the other quasi-equivalent interfaces, in both capsid morphologies. By these measures, the consensus ranking of the inter-dimer interfaces in terms of stability is AA > BC > CB in T = 3 capsids, and AA > BD > DC > CB in T = 4 capsids.

Analysis of the *intra*-dimer interfaces with *PDBePISA* also reveals differences between the four types of dimers present in T = 3 and T = 4 capsids (S7 Table). The T = 3 CC intra-dimer interface has a free energy less than the other three. Relative to the T = 4 CD dimer this amounts to *ca*. 10 kCal/mol. Mutual accommodation at the intra-dimer interface between chains in the C conformation may pose an energy barrier to formation of the CC dimer.

### Visualization of early assembly intermediates by negative stain electron microscopy

**Fig 8:**
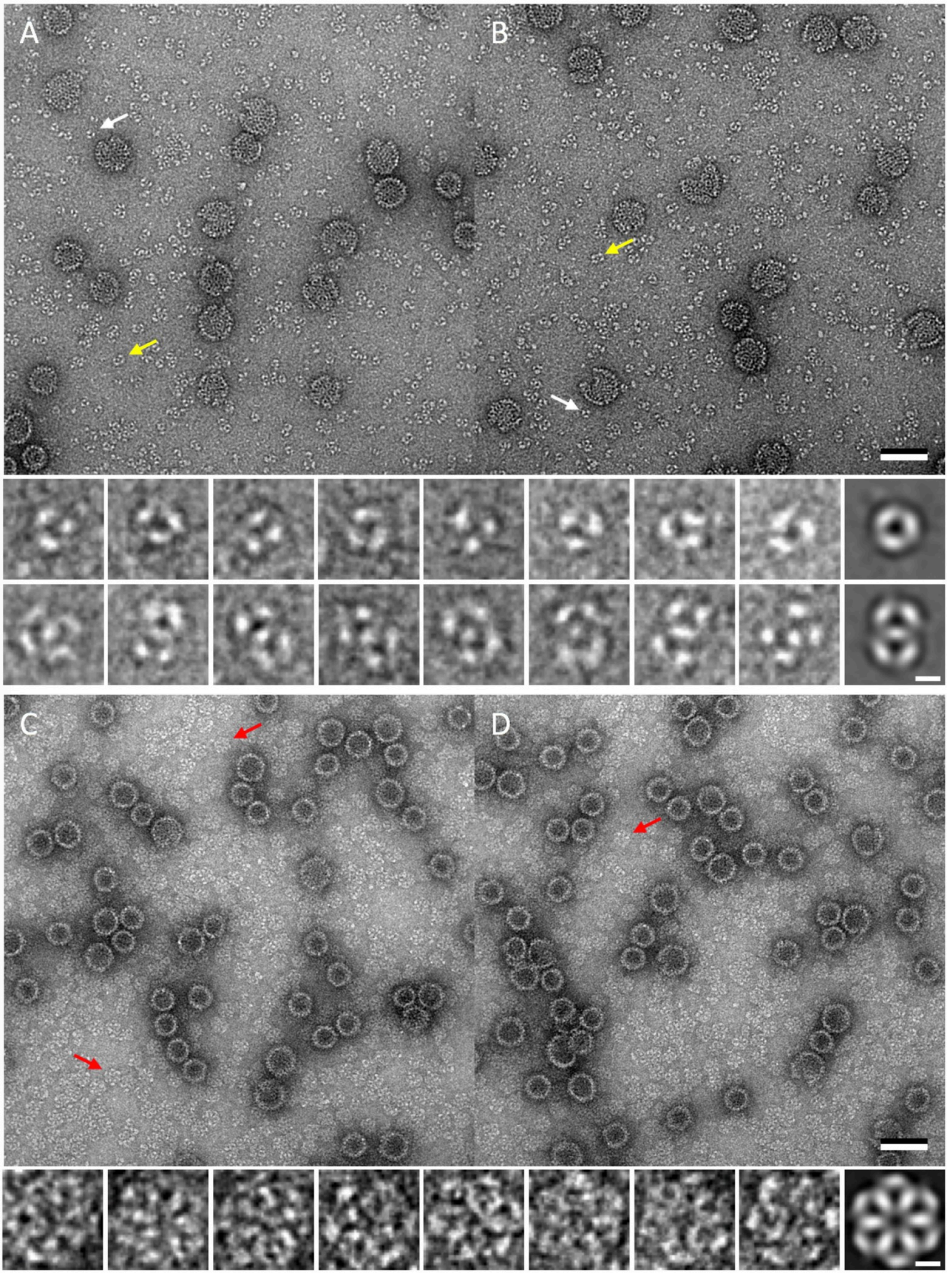
Products of assembly reactions as visualized by negative stain electron microscopy. (A, B) Under mild assembly conditions, core-antigen dimers form small complexes that appear to be trimers-of-dimers (white arrows) and pentamers-of-dimers (yellow arrows). Enlargements and class averages (right) are shown below. (C, D) At later times, six-fold symmetric structures (red arrows) that appear to consist of 12 dimers are formed. Enlargements and a class average (right) are shown below. Under non-assembly conditions, no structures are formed. Bar = 50 nm on main panels, 5 nm on enlargements.

Capsid assembly has been proposed to begin with the formation of a trimer-of-dimers nucleus and then to proceed by the further addition of dimers [13,14]. Early intermediates consisting of up to five dimers (with species consisting of three and five dimers prominent) have been detected by charge detection mass spectrometry (CDMS) [16] but have yet to be visualized. To visualize early intermediates, we examined reaction products formed after initiating assembly under mild conditions (low ionic strength and neutral pH; see Materials and Methods) by negative stain electron microscopy. We observed triangular complexes suggestive of trimers-of-dimers; larger, twofold-symmetric complexes that we interpret as pentamers-of-dimers; much less frequently, fivefold-symmetric complexes, apparently also pentamers of dimers; and at later time-points, sixfold-symmetric complexes that appear to consist of 12 dimers (Fig 8). Under assembly-negative conditions, no such complexes were observed (see also [42]). The formation of these complexes was sensitive to protein concentration, buffer conditions, and timing of sampling. The trimers-of-dimers and twofold-symmetric pentamers-of-dimers were formed in abundance (*ca*. 43% and 56%, respectively, of 484 picked particles), but the fivefold-symmetric pentamers-of-dimers were always rare (*ca*. 1%), suggesting that the latter are not early intermediates.

### Phylogeny of the Hepadnaviridae and capsid dimorphism

**Fig 9:**
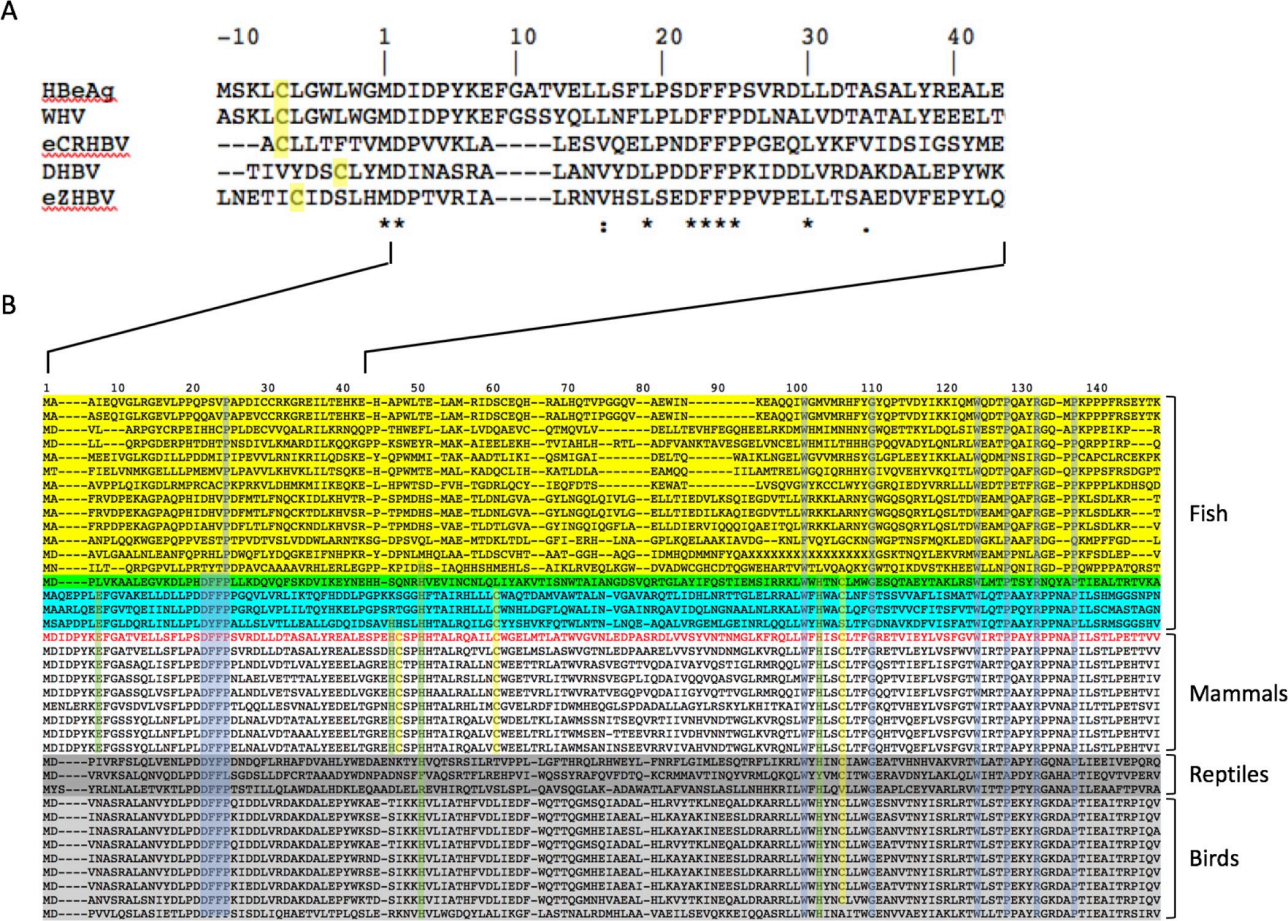
Multiple sequence alignment of core-antigen related proteins. (A) PreC/C ORF sequences. Shown are sequences for human (HBeAg), woodchuck (WHV), and duck (DHBV) viruses, and for zebra finch (eZHBV) and crocodile (eCRHBV) endogenized viral elements. For brevity, sequences are only shown up to the end of helix 2, in human core-antigen this is reside E43. (B) Core-antigen sequences. Sequence numbers correspond to human core-antigen residues 1–149. Viral clades are colored according to their host taxa: Nackednaviruses (yellow), Parahepadnaviruses (green), Metahepadnaviruses (cyan), Orthohepadnaviruses (none, except human ayw D, red), Herpetohepadnaviruses (dark gray), Avihepadnaviruses (light gray). There are seven residues (not counting M1) that are over 95% conserved in viruses from all four taxa of hosts (fish, mammals, reptiles, and birds). Shaded in the alignment are; the highly conserved residues (P25, W102, G111, W125, P129, R133, P138 and the DFFP motif at residues P22 –P25; light blue), the putative metal-binding motif (E8, H47, H51, and H104; light green), and the cysteine motif (C48, C61 and C107; light yellow). The sequences are adapted from previous phylogenetic analyses, as describe in text, by deleting insertions relative to the human sequence.

Quasi-equivalence enables the formation of capsids with triangulation numbers greater than 1. In the case of HBV, it also allows for dimorphism. The question arises, what aspect of the amino acid sequence determines dimorphism? Cryo-EM reconstruction of an African cichlid Nackednavirus revealed the characteristic fold and dimeric structure of the Hepadnaviral capsid protein, and its arrangement into only a T = 3 lattice [37]. Three copies of a predicted short helix (α+) immediately N-terminal to the start of the assembly domain in mammalian virus capsid proteins were found positioned around the three-fold axis of symmetry [37]. This is reminiscent of the T = 3 capsids formed by reduced e-antigen where three copies of the propeptide were positioned around the three-fold symmetry axis [28]. Our recent X-ray crystallographic structure of the e-antigen has shown the propeptide to have a helical structure [42]. It may be that the presence of three helices closely packed at this location induces greater curvature of early intermediates in assembly, favoring the formation of T = 3 capsids. Multiple sequence alignment of the preC/C ORFs in three extant Hepadnaviruses and two endogenized viral elements [37] revealed sequences similar to the human e-antigen propeptide in rodents but different in the avian and reptilian viral sequences (Fig 9A). The reptilian viral sequence shares the CL sequence at residues -7 and -6 and is also hydrophobic. The zebra finch viral sequence has ICI versus LCL but the C is at the -6 position. The Cysteine residue at the (-7) position in the propeptide is required for the formation of an intra-molecular disulfide bond with C61 to maintain the e-antigen dimer in its characteristic conformation [11,27,42]. These similarities suggest the early stages in the evolution of the e-antigen propeptide. The acquisition of the surface antigen ORF, by the process of overprinting of the reverse transcriptase coding sequence, may have necessitated the switch to the larger T = 4 capsid [37]. Placement of the selective presence of the propeptide under transcriptional control may have been involved in this process.

Multiple sequence alignment of Hepadnaviral capsid proteins from a wide range of host taxa (mammals, birds, reptiles, and diverse families of fishes) [36,37] reveals both clear similarities and differences within and between clades (Fig 9B). Some residues are very highly conserved. Seven of these residues (P25, W102, G111, W125, P129, R133, and P138) are 95% conserved and five are >97% conserved over all host taxa. That these five to seven residues have been so highly conserved for over 400 million years attests to their structural importance. All these residues, except G111, are located in the interaction region of the assembly domain, and they are arranged closely around the five- and two-fold symmetry axes in the capsids (S3 Fig A and B). None are located in the regions corresponding to the spike apices or immunodominant loops in capsids. These residues also correspond to the three domains identified in T = 4 capsids by solid state NMR [9] namely: (i) the turn between helices 1 and 2, (ii) the base of helix 4b, and (iii) helix 5 and the C-terminal strand (S3 Fig C). They also correspond to the dynamic domains identified in T = 4 capsids [7] and with *DynDom* in the T = 3 and T = 4 capsids described here. There are also striking differences between the clades, particularly between the Nackednaviruses and all others. The distinctive motif DFFP (residues 22–25), perfectly conserved in the mammalian and avian viral sequences, and mostly so in the reptilian and enveloped fish viral sequences (Fig 9B), corresponds to the “fulcrum” region between helices 1 and 2 [7,36] and also the region identified in the *DynDom* analysis as moving in concert with the C-terminal domain (Fig 4C). This motif is completely absent in the Nackednaviruses. Should the capsids from all Hepadnaviral clades except the Nackednaviridae turn out to be dimorphic, then the coincidence of this motif with the transition from monomorphism would suggest a role in symmetry regulation. Taken together, the results above indicate that regulation of dimorphism occurs on two levels; in an evolutionary sense with the loss of the α+ helix and gain of the DFFP motif, and in extant viruses at the transcriptional level with the selective expression of the preC/C region.

## Discussion

We have visualized the T = 3 and T = 4 capsids at high resolution (3.5–3.6 Å) under identical solution and imaging conditions, thereby allowing direct and quantitative comparison of their structures. The seven quasi-equivalent chains have different conformations and potential energies, with the T = 3 C chain having the lowest, suggesting it is the least strained. Three of the four quasi-equivalent dimers are asymmetric with respect to conformation and potential energy, however the T = 3 CC dimer is symmetrical and has the lowest potential energy while its intra-dimer interface has the least free energy of formation. The CB inter-dimer interface has the least area and free energy, in both morphs. Trimers-of-dimers and pentamers-of-dimers are discernible in the capsid lattices, and their status as putative assembly intermediates is supported by their predominance among the assembly products observed by negative stain electron microscopy. The three kinds of trimers-of-dimers and four kinds of pentamers-of-dimers vary in their potential energies, with some having positive values, suggesting they are under strain.

To interpret these results, we first recall some earlier observations. It has been proposed that capsid assembly is nucleated by formation of a trimer-of-dimers, to which further dimers are subsequently added [13]. Intermediates between dimers and fully assembled capsids are short-lived species [13,16,17] but nevertheless early intermediates have been identified by CDMS. Under mild assembly conditions similar to those employed here, CDMS peaks corresponding to species consisting of one, two, three, and five dimers have been observed [16,17]. The next larger intermediates detected correspond to complexes of *ca*. 90 dimers [65]. What occurs between these two states, including the point(s) where the paths leading to T = 3 and T = 4 capsids diverge, has been obscure though progress is being made [66]. It has been reported that T = 3 capsids form early and briefly (seconds) whereas T = 4 capsids continue to be assembled for extended periods (hours), eventually becoming the majority species [67]. When capsids were repeatedly dissociated with 2 M urea and reassembled, the proportion of T = 3 capsids increased with each cycle [68]. Finally, when capsids were held under low ionic strength conditions for extended periods in the cold, only the T = 3 capsids exchanged subunits with those in solution, with the number exchanged suggesting an origin over the twofold axis [68]. How might these various observations be explained in view of the present findings?

HBV capsids may be viewed as being built from fivefold-symmetric AB pentamers linked either by CC dimers (T = 3 capsids) or by Type II trimers-of-dimers (T = 4 capsids). Alternatively–and more appropriately for subassemblies–they may be viewed as being composed of trimers-of-dimers, with T = 3 capsids consisting of only one form (Type I) and T = 4 capsids of two forms (Type I and Type II) (Figs 2 and 7C). Assembly of T = 3 capsids depends on the availability of CC dimers. When the supply of CC dimers is exhausted, T = 3 assembly stops. The observation that C chains, and CC dimers, have substantially lower potential energies than their counterparts (Fig 7A and 7B) suggests an energy barrier between these conformations, potentially involving the CC intra-dimer interface (S7 Table). Exposure to 2 M urea may permit more dimers to cross the barrier and adopt the lower energy CC conformation, thereby promoting the assembly of T = 3 capsids. During cold disassembly, the CC dimers, having two weak hydrophobic (CB) interfaces with the rest of the lattice (S4 Fig and Table 4), are likely to dissociate preferentially [68]. If the AB dimers of the two capsids are sufficiently alike (Fig 2), then the point where the T = 3 and T = 4 assembly pathways diverge would be the formation of either a CC or a CD dimer. These dimers can only go on to form either T = 3 or T = 4 capsids, respectively. In T = 4 capsids there are two forms of trimer-of-dimers, Types I and II. As Type I has the lower potential energy (Fig 7C), this form should persist and nucleate T = 4 capsid assembly for protracted periods [67]. Adding two dimers to the same facet of a trimer-of-dimers gives a pentamer-of-dimers, putatively the next on-pathway intermediate. In T = 3 capsids, the Type I form has lower energy and is therefore likely to be the next intermediate, whereas in T = 4 capsids, the Type II form has lower energy, and this is probably the next intermediate (Fig 7D).

Our negative staining EM experiments did not detect any clearcut intermediates between the twofold-symmetric pentamers-of-dimers and sixfold-symmetric complexes of 12 dimers (Fig 8C and 8D). This may mean that the latter are formed by antiparallel joining of two T = 4 Type II pentamers-of-dimers *via* the addition of just two more low energy CD dimers (Fig 2). This kind of growth is not possible for T = 3 capsids (Fig 2), suggesting that the observed sixfold-symmetric complexes are intermediates in T = 4 capsid assembly. In summary, we propose that (i) the pathway to T = 3 capsids involves AB and CC dimers, T = 3 Type I trimers-of-dimers, and T = 3 Type I pentamers-of-dimers, followed by the addition of further dimers, and (ii) the pathway to T = 4 capsids involves AB and CD dimers, T = 4 Type I trimers-of-dimers, T = 4 Type II pentamers-of-dimers, joining of the latter by two CD dimers, followed by the addition of further dimers. Unlike the assembly of core-antigen capsids, which assemble mostly with T = 4 symmetry, reduction of the intramolecular disulfide of the e-antigen results in formation of capsid-like structures with T = 3 symmetry [28]. In this case, the symmetry is probably driven by greater curvature of early intermediates due to the crowding of three copies of the propeptide around the threefold symmetry axis [28], analogous to the presence of the N-terminal α+ helix at this location in the T = 3 capsids of Nackednaviruses [37].

Interpretation of our results in terms of early intermediates on the two capsid assembly pathways is subject to several qualifications. Likely the most important of these is the limited resolution of the two reconstructions, primarily due to the inherent flexibility of the capsids. Resolution limits the accuracy with which models can be built into the electron density maps, which in turn limits estimation of their potential energy (a function of all the bonded and non-bonded interactions of all atoms in the system; essentially the enthalpies). Perhaps next most important is the question of whether the potential energies of two polypeptide chains (and their assemblies) can legitimately be compared. While there can be no comparison between chemically different polypeptide chains, and also no comparison between chemically identical chains in unrelated environments, it is reasonable to qualitatively compare chemically identical chains when they have quasi-equivalent conformations in quasi-equivalent environments, and they have been energy minimized with the same force field. Also important is the role of entropy as capsid assembly is driven by a decrease in free energy (enthalpy and entropy) of the entire system (protein and solution). HBV capsid assembly is driven by hydrophobic interactions and therefore the entropy of water plays an important role. However, changes in solution entropy during the assembly process cannot be determined by cryo-EM. The conformational entropy of the protein before, during, and after assembly also changes. For example, dimers before assembly have translational and rotational degrees of freedom that do not exist in the assembled state. Our reconstructions only assess the fully assembled state. Another problem is detecting conformational entropy and discriminating it from other contributions. In single particle analysis reconstruction, unlike crystallography, individual particles must be imaged, identified, aligned, and averaged. In the case of capsids with icosahedral symmetry this is also applied. The quality of the resulting density map is given as the global resolution. However, the resolution can vary spatially due to flexibility (conformational entropy) but also due to different conformational states, partial occupancy in the particles, as well as non-isotropic views, such as due to interactions with the air-water interface prior to freezing. This variation is given as local resolution, an order parameter analogous to the crystallographic B-factor [69]. To measure local conformational entropy differences (e.g. on either side of a dimer) experimentally in capsids it would be necessary to extract it from local resolution. How to do so is not clear. How to measure local conformational entropy differences experimentally in early intermediates, particularly those that are fleeting, is also not clear. Therefore, at present, it is not possible to assign symmetry-specific entropic contributions by cryo-EM and so we provisionally assume them to be similar.

### Summary

By fractionating and remixing the T = 3 and T = 4 capsids in equal numbers, by recording data under identical solution and imaging conditions, by processing both datasets in the same manner, and by performing energy minimizations on models of the intact capsids and the extracted subassemblies with the same force field, it was possible to directly and quantitatively compare the potential energies of the seven quasi-equivalent chains, the four types of dimers, the three types of trimers-of-dimers, and the four types of pentamers-of-dimers identifiable in the capsid lattices. The potential energy values of such structures are an accurate measure of their conformational differences, even while those values do not represent free energies. By comparing all substructures from both capsids in the same way, and by assuming entropic contributions to be similar between quasi-equivalent sites, we can obtain insights into capsid assembly, symmetry determination, and disassembly that at present are otherwise experimentally inaccessible.

## Materials and methods

### Specimen preparation

The Hepatitis B virus capsid protein assembly domain construct Cp149.3CA, i.e. residues 1–149 but missing the C-terminal nucleic acid-binding tract of the full-length protein, and in which all three cysteine residues have been mutated to alanine (including C61 which can form an inter-molecular disulfide bond), was expressed in *Escherichia coli* and purified as described previously [55]. Protein was stored frozen in 100 mM sodium carbonate (pH 9.6) at -80°C. The capsids were assembled by overnight dialysis against 100 mM Tris (pH 7.5), 300 mM NaCl at room temperature. The resulting mixture of T = 3 and T = 4 capsids (*ca*. 5% T = 3 and 95% T = 4) was resolved on a 5–30% sucrose gradient in 50 mM Tris (pH 7.5), 150 mM NaCl. Gradients were formed by tilted-tube rotation (time, angle, speed; 1:36, 81.5°, 19 rpm) (BioComp) and centrifuged at 284,000 xg for 2 h at 20°C in a Beckman SW40 Ti rotor. The gradients were high-resolution fractionated by piston displacement (BioComp) and the T = 3 and T = 4 capsids were collected, dialyzed overnight against 50 mM Tris (pH 7.5), 150 mM NaCl at room temperature and concentrated to 3 mg/ml by centrifugal ultrafiltration (Amicon Ultra 4). The two preparations were checked for purity, particle condition, and dimorphism by negative stain electron microscopy in 1% uranyl acetate. Images were recorded at 35,000x nominal magnification on a CCD with a CM120 electron microscope (FEI) and particles counted. The T = 3 preparation consisted of *ca*. 85% T = 3 particles, and the T = 4 preparation consisted of *ca*. 94% T = 4 particles. Thereafter the preparations were mixed in volume proportions to give 50% of each symmetry.

To observe capsid assembly intermediates, Cp149.3CA in 100 mM sodium carbonate (pH 9.6) was diluted to 0.1 mg/ml with mild (low ionic strength) assembly buffers (10 mM Tris (pH 7.5), 10–100 mM NaCl) and incubated at room temperature for times ranging from five seconds up to 1 minute (in 5 second intervals) and then at 5, 10, 15, 30 and 60 minutes before applying to glow-discharged, 0.1% poly-lysine coated carbon grids and staining with 1% uranyl acetate. Images were recorded as described above.

### Data collection

3-μl drops were applied to R1.2/1.3 Quantifoil 400 mesh copper grids (EMS), blotted, and plunge-frozen in liquid ethane using a Reichert KF-80 cryostation (Leica). 339 dose fractionation series were collected using a Polara electron microscope equipped with a K2 camera (300 kV, 20,000x nominal magnification [40,000x in super-resolution mode], 1–2 μm defocus, 33 frames/series, 10 electrons/pixel/second exposure, 25 e^-^/Å^2^ total exposure, 1.015 Å/pixel). Each series was averaged using *MotionCor2* [70]. The particles were picked semi-automatically from the same averaged micrographs using *e2boxer*.*py* [71], first the T = 3 and then the T = 4. In total, there were *ca*. 15,000 T = 3 particles and 14,000 T = 4 particles, with image sizes of 432 and 480 pixels, respectively. The final pixel size was corrected to 0.93 Å/pixel.

### Image analysis

*Relion* [72] was used to process the T = 3 and T = 4 datasets by following the standard protocol, but with symmetry I4, meaning the output map had a fivefold Z-axis, consistent with the default in *EMAN/EMAN2* [71]. The output.star files were then converted to list files, together with the two output maps (each map from a half-dataset) and treated as input files for further refinement in EMAN2 using *jspr/jalign*. Further refinement using *jspr/jalign* included orientation refinement, per-particle level defocus refinement, scale refinement, astigmatism refinement, and beam-tilt refinement. Three iterations were sufficient to optimize the results and obtain final maps. The resolution was determined from both the FSC curves and real features to be 3.5 and 3.6 Å for the T = 3 and T = 4 capsids, respectively.

For assembly intermediates visualized by negative stain electron microscopy, class averages were calculated with Bsoft [73]. No symmetry was imposed on the class averages of the presumptive trimers-of-dimers and pentamers-of-dimers, however, six-fold symmetry was applied to the hexameric complexes apparently composed of twelve dimers.

### Model building

The X-ray crystal structure of the HBV T = 4 capsid (PDB ID: 1QGT) was rigid-body fitted into the T = 3 and T = 4 density maps using *UCSF Chimera* [60] to find the initial positions; per convention, the A chain was positioned in the penton region. Each chain was then fitted individually into the density maps to obtain an optimal fit (for T = 4, the A, B, C, and D chains were used; for T = 3, the A, B, and C chains were used). The fitting results were then used as a template in *Phenix* [74] for refinement on those T = 3 and T = 4 maps to obtain the T = 3 and T = 4 capsid atomic models. The full capsid structures were then globally energy minimized with force field AMBER ff14SB (the default) to obtain the final models. The Electron Microscopy Databank entries for the T = 3 and T = 4 capsid density maps are EMD-20669 and EMD-20670, and the corresponding PDB accession codes are 6UI6 (see S1 Appendix) and 6UI7 (see S2 Appendix), respectively.

### Structure assessment

The quality of the structural models was assessed with *MolProbity* [52]. Domain dynamics were analyzed with *DynDom* [59]. Energy minimization was done with *MMTK* (as a tool within *UCSF Chimera*) using the AMBER force field ff14SB [60]. The potential energy calculations were performed as follows. The seven types of single chains, four types of dimers, three types of trimers-of-dimers, and four types of pentamers-of-dimers were extracted from the full T = 3 and T = 4 capsid models and then subjected to further energy minimization in isolation. Hydrogens and charges were added using defaults in *UCSF Chimera*. Waters and counterions were not included. Steepest descent energy minimization curves (rather than just single values) were plotted in order to better represent the relationships between the structures. For surveys, the structures were minimized for 1,000 cycles with the data averaged every ten cycles. For the more extensive analyses, the structures were minimized for 10,000 cycles with the data averaged every ten cycles. The initial (single cycle) potential energy values for the structures varied widely and therefore minimization curves were plotted beginning with the first ten-told averaged value. Following steepest descent energy minimization, conjugate gradient minimization was also performed, however, as this resulted in only a minimal change in the final values obtained the results were not included in the final analysis. Protein interfaces were examined with *PDBePISA* [75]. Interface computational alanine scanning was performed with *KFC2* [63,64]. Interface regions were visualized with the Fast Atomic Density Evaluation (*FADE*) algorithm on the *KFC2* server. Molecular illustrations were prepared with *UCSF Chimera* [60].

## Supporting information

S1 Fig Ribbon diagrams of core-antigen polypeptide chains colored by Cα-RMSD of T = 3 and T = 4 chains aligned to the T = 3 C chain. The chains are colored minimum-maximum, Blue-Red. The color range is normalized across all panels (RMSD: 0 to 6.64 Å with white set at 0.5 Å, i.e. at the middle of the histogram peak). The T = 3 C chain is not shown as it aligns perfectly to itself. Helices are numbered in (A). The core region shows the least difference and the C-terminal region the most difference.
(TIFF)

S2 Fig The seven quasi-equivalent interfaces in T = 3 and T = 4 capsids. (A) The interface region (Cyan) between two chains. (B) Three orthogonal views of the interface regions. Rotations in the second and third rows relative to the first row are indicated on the left. The volumes were calculated with *FADE* and represent the space between the two chains, 3 Å from each. In both T = 3 and T = 4 capsids the CB interface is the smallest.
(TIFF)

S3 Fig Location of the highly-conserved residues in the context of the capsid. (A) Outside and (B) inside views of the capsid. All the highly conserved residues identified in Fig 9 are clustered closely in the interaction region of the assembly domain, and they are arranged around the 5- and 2-fold (but not 3-fold) symmetry axes. (C) Ribbon diagram of two monomers with the conserved residues highlighted in red.
(TIFF)

S4 Fig T = 3 capsid disassembly. The CB interface is the weak point in capsids. As shown in Table 4, the calculated free energy of the T = 3 CB interface is *ca*. 2 kCal/mol (20%) lower than the mean of the other interfaces. Unlike T = 4 capsids, T = 3 capsids have CC dimers, each of which has *two* low-affinity interactions with the surrounding dimers (arrows), allowing them to more easily dissociate from the lattice. The monomers are colored according to the conventional scheme: A, green; B yellow; C, red.
(TIFF)

S1 Table HBV core-antigen related structures in the EMDB and PDB databases^1^. ^1^ Only human viral structures are listed, i.e. no WHV, although the piscine T = 3 Nackednaviral structures are included as they are considered in this study. Also, only core-antigen structures are included, i.e. no e-antigen. ^2^ 3J2V, 6BVF, and 6BVN are in both databases but are only shown once here, in the PDB. ^3^ Symmetry; NA indicates a non-capsid complex. ^4^ When no resolution was reported (-).
(DOCX)

S2 Table Comparison of the quality of structures in this study with reference structures. This table is similar to Table 1 but is more inclusive. ^1, 2^ All score values are percent (%) except Clash score and *Molprobity* score, which are percentile, as defined below. ^3^ Clash score is the number of serious steric overlaps (>0.4 Å) per 1,000 atoms. ^4^ *Molprobity* score combines clash, rotamer, and Ramachandran evaluations into a single score, normalized to be on the same scale as X-ray resolution. For both Clash score and *Molprobity* score the values are percentile (100^th^ is best, 0^th^ is worst) relative to a set of comparable structures determined for each calculation (see *Molprobity* server for details). Analysis performed with PROCHECK confirmed the quality of the current structures.
(DOCX)

S3 Table All-atom RMSD (Å) of chain-pairs in select core-antigen structures^1^. ^1^ All structures are T = 4 capsids except 3KXS, which is a core-antigen dimer complex. All structures are apo, i.e. non-liganded.
(DOCX)

S4 Table Conformational analysis of chain pairs with Dyndom^1^. ^1^ All the chain pairs in the table were analyzed with *DynDom*. However, when the chain pairs did not meet the specific requirements of being “dynamic”, even if their conformations were different, then no values were returned by the program and the cells were left blank. ^2^ Indicates that chains from T = 3 capsids were compared to chains from T = 4 capsids, in the order given.
(DOCX)

S5 Table Computational alanine scanning interface analysis of T = 4 capsids^1^. ^1^ Results from a *KFC2* computational alanine scanning interface analysis of the four quasi-equivalent sites (AA, BD, CB, and DC) in the T = 4 capsids described in this study. Residues identified as hot spots by *KFC2* are listed separately for each chain in a dimer. Residues in bold font were classed as high confidence by *KFC*2. Area and volume values are for the interface region between the two chains, 3 Å from each chain, as calculated with *FADE* (on the *KFC2* server) and *UCSF Chimera*. Note that the CB interface is smaller than the other three and that V124 and R127 are not classed as part of the CB interface (boxed cells).
(DOCX)

S6 Table Computational alanine scanning interface analysis of T = 3 capsids^1^. ^1^ Results from a *KFC2* computational alanine scanning interface analysis of the three quasi-equivalent sites (AA, BC, and CB) in the T = 3 capsids described in this study. Residues identified as hot spots by *KFC2* are listed separately for each chain in a dimer. Residues in bold font were classed as high confidence by *KFC2*. Area and volume values are for the interface region between the two chains, 3 Å from each chain, as calculated with *FADE* (on the *KFC2* server) and *UCSF Chimera*. Note that the CB interface is smaller and that V124 and R127 are not classed as part of the CB interface (boxed cells).
(DOCX)

S7 Table *PISA* intra-dimer interface analysis of T = 3 and T = 4 capsids. (DOCX)

S1 Appendix PDB/EMDB validation report for T = 3 capsid (PDB: 6UI6). (PDF)

S2 Appendix PDB/EMDB validation report for T = 4 capsid (PDB: 6UI7). (PDF)
