# Supplementary figures and images for: Expression of quasi-equivalence and capsid dimorphism in the Hepadnaviridae
Source: PLoS Comput Biol. 2020 Apr 20;16(4):e1007782. doi: 10.1371/journal.pcbi.1007782 (PMC7192502; doi:10.1371/journal.pcbi.1007782)

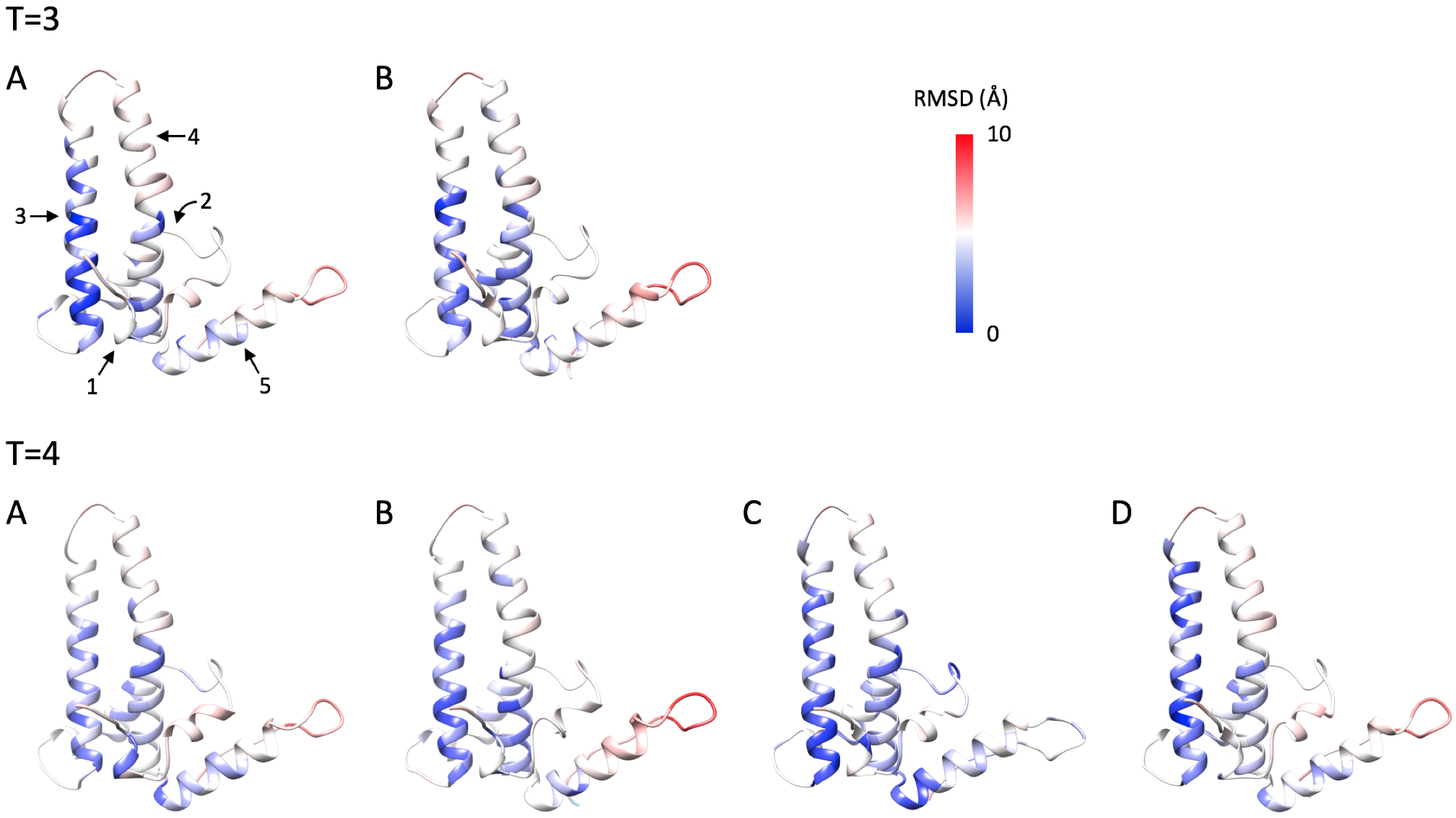

Supplement: S1 Fig — The chains are colored minimum-maximum, Blue-Red. The color range is normalized across all panels (RMSD: 0 to 6.64 Å with white set at 0.5 Å, i.e. at the middle of the histogram peak). The T = 3 C chain is not shown as it aligns perfectly to itself. Helices are numbered in (A). The core region shows the least difference and the C-terminal region the most difference. (TIFF) [file pcbi.1007782.s001.tiff]

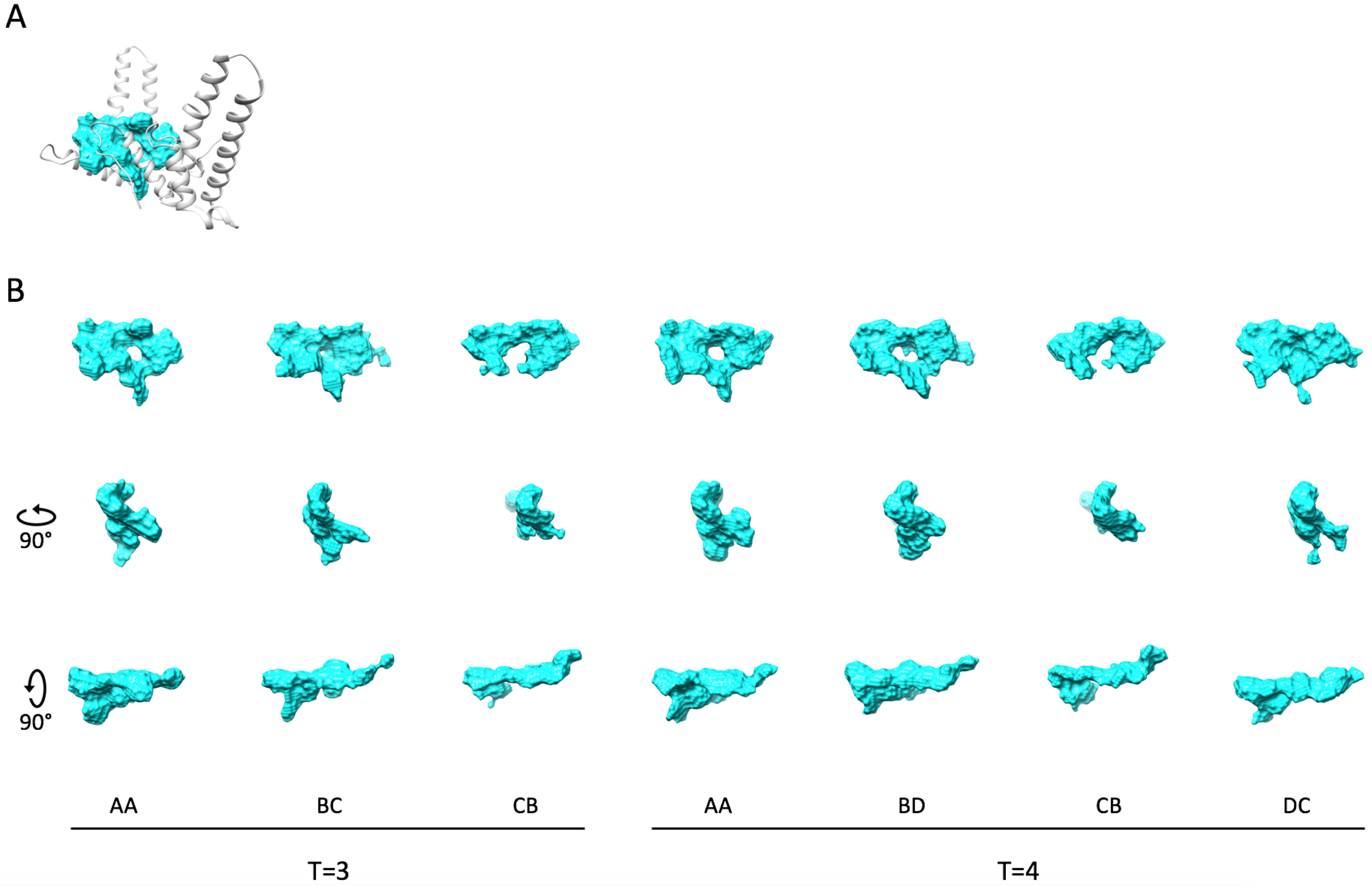

Supplement: S2 Fig — (A) The interface region (Cyan) between two chains. (B) Three orthogonal views of the interface regions. Rotations in the second and third rows relative to the first row are indicated on the left. The volumes were calculated with FADE and represent the space between the two chains, 3 Å from each. In both T = 3 and T = 4 capsids the CB interface is the smallest. (TIFF) [file pcbi.1007782.s002.tiff]

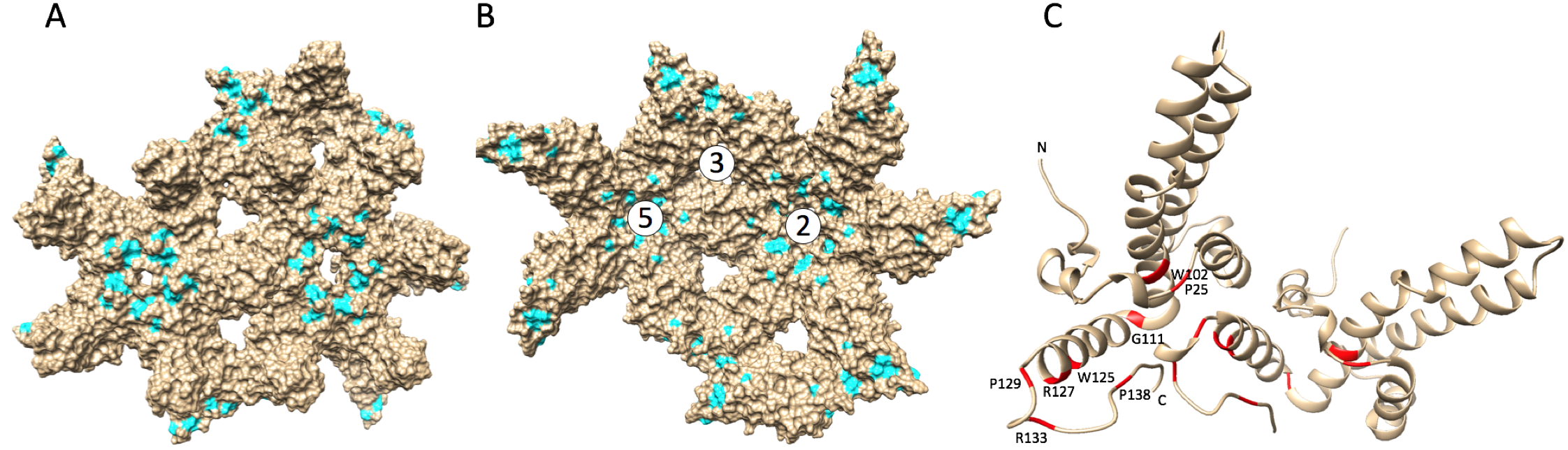

Supplement: S3 Fig — (A) Outside and (B) inside views of the capsid. All the highly conserved residues identified in Fig 9 are clustered closely in the interaction region of the assembly domain, and they are arranged around the 5- and 2-fold (but not 3-fold) symmetry axes. (C) Ribbon diagram of two monomers with the conserved residues highlighted in red. (TIFF) [file pcbi.1007782.s003.tiff]

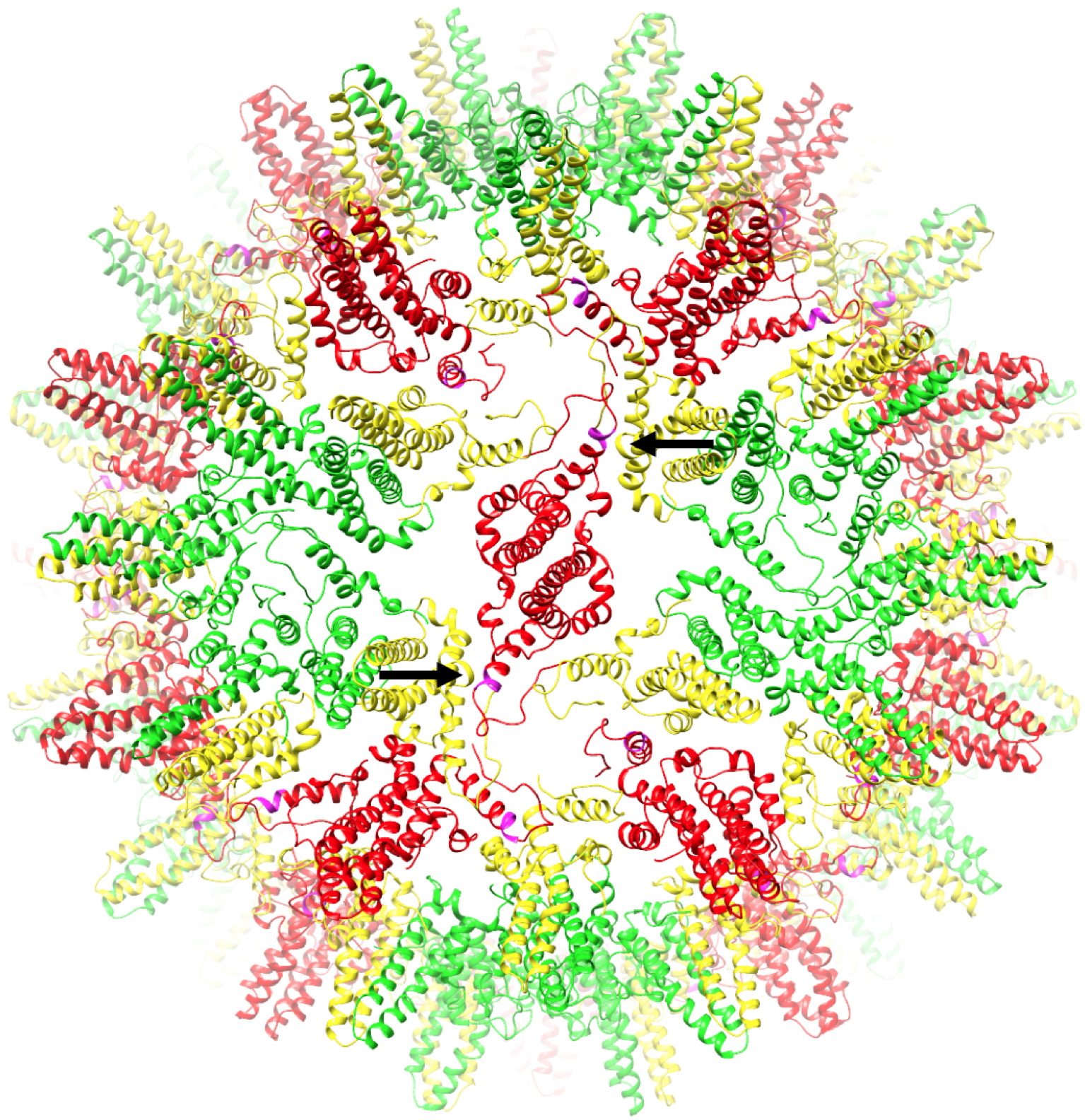

Supplement: S4 Fig — The CB interface is the weak point in capsids. As shown in Table 4, the calculated free energy of the T = 3 CB interface is ca. 2 kCal/mol (20%) lower than the mean of the other interfaces. Unlike T = 4 capsids, T = 3 capsids have CC dimers, each of which has two low-affinity interactions with the surrounding dimers (arrows), allowing them to more easily dissociate from the lattice. The monomers are colored according to the conventional scheme: A, green; B yellow; C, red. (TIFF) [file pcbi.1007782.s004.tiff]
