# Supplementary material for: Expression of quasi-equivalence and capsid dimorphism in the Hepadnaviridae
Source: PLoS Comput Biol. 2020 Apr 20;16(4):e1007782. doi: 10.1371/journal.pcbi.1007782 (PMC7192502; doi:10.1371/journal.pcbi.1007782)
Supplement: S1 Table — 1 Only human viral structures are listed, i.e. no WHV, although the piscine T = 3 Nackednaviral structures are included as they are considered in this study. Also, only core-antigen structures are included, i.e. no e-antigen. 2 3J2V, 6BVF, and 6BVN are in both databases but are only shown once here, in the PDB. 3 Symmetry; NA indicates a non-capsid complex. 4 When no resolution was reported (-). (DOCX) [file pcbi.1007782.s005.docx]

**S1 Table. HBV core-antigen related structures in the EMDB and PDB Databases.^1^**

| EMDB | PDB^2^ | Symmetry^3^ | Method | Resolution (Å)^4^ | Comment |
| --- | --- | --- | --- | --- | --- |
| 1399 |  | T=4 | EM | - | Capsid-envelope contacts |
| 1400 |  | T=4 | EM | 22 | “ |
| 1401 |  | T=4 | EM | 22 | “ |
| 1402 |  | T=4 | EM | 22 | “ |
| 1403 |  | T=4 | EM | - | “ |
| 1404 |  | T=4 | EM | - | “ |
| 1405 |  | T=4 | EM | - | “ |
| 1406 |  | T=4 | EM | - | “ |
| 1407 |  | T=4 | EM | - | “ |
| 1408 |  | T=4 | EM | - | “ |
| 1968 |  | T=4 | EM | 14.2 | Capsid_183 SRPK complex |
| 1969 |  | T=4 | EM | 17.4 | Capsid_183 |
| 2046 |  | T=4 | EM | 10.1 | Capsid_E1 Ab complex |
| 2057 |  | T=4 | EM | 5.5 | Capsid_183 |
| 2058 |  | T=4 | EM | 5.8 | Capsid_183 phosphorylation |
| 2059 |  | T=4 | EM | 8.0 | Capsid_183 pgRNA |
| 2060 |  | T=4 | EM | 7.0 | Capsid_183 |
| 2509 |  | T=4 | EM | 14.5 | Capsid (immature) |
| 3015 |  | T=4 | EM | 8.1 | Capsid single-tomogram averaging |
| 3266 |  | T=4 | EM | 10.1 | Capsid |
| 3267 |  | T=4 | EM | 10.9 | Capsid Importin-ß complex |
| 3268 |  | T=4 | EM | 10.2 | “ |
| 3269 |  | T=4 | EM | 10.1 | “ |
| 3270 |  | T=4 | EM | 13.9 | “ |
| 3271 |  | T=4 | EM | 8.9 | “ |
| 3272 |  | T=4 | EM | 15.9 | “ |
| 3714 |  | T=4 | EM | 11.4 | Capsid RNA |
| 3715 |  | T=4 | EM | 4.7 | Capsid VLP |
| 3716 |  | T=3 | EM | 5.6 | Capsid VLP |
| 3822 |  | T=3 | EM | 8.0 | Capsid of Nackednavirus |
| 3823 |  | T=3 | EM | 9.0 | Capsid of Nackednavirus |
| 6887 |  | T=4 | EM | 6.6 | To be published |
| 6888 |  | T=4 | EM | 6.4 | To be published |
|  | 1QGT | T=4 | X-ray | 3.4 | Capsid (apo) |
|  | 2G33 | T=4 | X-ray | 3.96 | Capsid (apo) |
|  | 2G34 | T=4 | X-ray | 3.96 | Capsid with Hap1 ligand |
|  | 2QIJ | T=4 | X-ray | 8.9 | Capsid N-terminal extension |
|  | 3J2V | T=4 | EM | 3.5 | Capsid (apo) |
|  | 3KXS | NA | X-ray | 2.25 | Core-dimer complex (apo) |
|  | 4BMG | NA | X-ray | 3.0 | Core dimer folding |
|  | 4G93 | T=4 | X-ray | 4.2 | Capsid with AT130 ligand |
|  | 5D7Y | T=4 | X-ray | 3.89 | Capsid with HAP18 ligand |
|  | 5E0I | NA | X-ray | 1.95 | Core dimer with NVR10-001E2 ligand |
|  | 5GMZ | NA | X-ray | 1.7 | Core dimer with 4-methyl HAP ligand |
|  | 5TP2 | NA | X-ray | 1.69 | Core dimer with SBA_R01 ligand |
|  | 5WRE | NA | X-ray | 1.95 | Core dimer with HAP_R01 ligand |
|  | 5WTW | NA | X-ray | 2.62 | Core dimer (apo) |
|  | 6BVN | T=3 | EM | 4.0 | Capsid with HAP-TAMRA ligand |
|  | 6BVF | T=4 | EM | 4.0 | Capsid with HAP-TAMRA ligand |
|  | 6CWD | NA | X-ray | 3.33 | Core dimer with scFv e13 |
|  | 6CWT | NA | X-ray | 3.15 | Core dimer with Fab e21 |

^1^ Only human viral structures are listed, i.e. no WHV, although the piscine T=3 Nackednaviral structures are included as they are considered in this study. Also, only core-antigen structures are included, i.e. no e-antigen.

^2^ 3J2V, 6BVF, and 6BVN are in both databases but are only shown once here, in the PDB.

^3^ Symmetry; NA indicates a non-capsid complex.

^4^ When no resolution was reported (-).
