# Supplementary material for: Expression of quasi-equivalence and capsid dimorphism in the Hepadnaviridae
Source: PLoS Comput Biol. 2020 Apr 20;16(4):e1007782. doi: 10.1371/journal.pcbi.1007782 (PMC7192502; doi:10.1371/journal.pcbi.1007782)
Supplement: S2 Table — This table is similar to Table 1 but is more inclusive. 1, 2 All score values are percent (%) except Clash score and Molprobity score, which are percentile, as defined below. 3 Clash score is the number of serious steric overlaps (>0.4 Å) per 1,000 atoms. 4 Molprobity score combines clash, rotamer, and Ramachandran evaluations into a single score, normalized to be on the same scale as X-ray resolution. For both Clash score and Molprobity score the values are percentile (100th is best, 0th is worst) relative to a set of comparable structures determined for each calculation (see Molprobity server for details). Analysis performed with PROCHECK confirmed the quality of the current structures. (DOCX) [file pcbi.1007782.s006.docx]

**S2 Table. Comparison of the quality of structures in this study with reference structures.**

| PDB code | 1QGT | 2G33 | 2G34 | 3J2V | 3KXS | 4G93 | 5D7Y | 5E0I | 6BVF | 6BVN | This study | |  |
| --- | --- | --- | --- | --- | --- | --- | --- | --- | --- | --- | --- | --- | --- |
| Capsid / Non-capsid | C | C | C | C | N | C | C | N | C | C | C | C |  |
| Apo / Liganded | A | A | L | A | A | L | L | L | L | L | A | A |  |
| Method | X-ray | X-ray | X-ray | EM | X-ray | X-ray | X-ray | X-ray | EM | EM | EM | EM |  |
| Resolution (Å) | 3.3 | 4.0 | 5.0 | 3.5 | 2.3 | 4.2 | 3.9 | 1.9 | 4.0 | 4.0 | 3.5 | 3.5 |  |
| R-value free | - | 0.372 | 0.342 | - | 0.264 | 0.383 | 0.273 | 0.252 | - | - | - | - |  |
| R-value work | 0.271 | 0.360 | 0.365 | - | 0.217 | 0.379 | 0.263 | 0.219 | - | - | - | - |  |
| Symmetry | T=4 | T=4 | T=4 | T=4 | - | T=4 | T=4 | - | T=4 | T=3 | T=3 | T=4 |  |
| Chains | ABCD | ABCD | ABCD | ABCD | ABCDEF | ABCD | ABCD | ABCDEF | ABCD | ABC | ABC | ABCD |  |
| Molprobity score | Score^1^ | | | | | | | | | | | | Goal^2^ |
| Clash (all atom)^3^ | 18^th^ | 16^th^ | 13^th^ | 97^th^ | 30^th^ | 40^th^ | 97^th^ | 98^th^ | 78^th^ | 89^th^ | 97^th^ | 99^th^ | - |
| Poor rotamers | 28.46 | 8.93 | 10.16 | 9.90 | 6.77 | 2.0 | 12.24 | 2.99 | 0.00 | 0.00 | 0.00 | 0.00 | <0.3 |
| Favored rotamers | 53.95 | 76.19 | 79.08 | 82.33 | 85.25 | 87.58 | 77.14 | 93.08 | 98.98 | 99.46 | 96.76 | 98.79 | >98 |
| Ramachandran outliers | 8.19 | 18.28 | 25.26 | 0.70 | 3.58 | 11.17 | 3.02 | 0.00 | 0.00 | 0.00 | 0.00 | 0.00 | <0.05 |
| Ramachandran favored | 66.73 | 55.17 | 45.30 | 95.45 | 90.21 | 57.66 | 88.08 | 96.46 | 92.14 | 95.54 | 95.25 | 94.66 | >98 |
| *Molprobity* score^4^ | 7^th^ | 30^th^ | 19^th^ | 97^th^ | 17^th^ | 67^th^ | 85^th^ | 81^th^ | 98^th^ | 100^th^ | 96^th^ | 97^th^ | - |
| Cß deviation (>0.025 Å) | 0.18 | 0.35 | 0.72 | 0.00 | 0.25 | 0.00 | 0.92 | 0.94 | 0.00 | 0.00 | 0.00 | 0.00 | 0 |
| Bad bonds | 0.00 | 0.00 | 0.00 | 0.00 | 0.03 | 0.06 | 0.04 | 0.04 | 0.00 | 0.00 | 0.00 | 0.00 | 0 |
| Bad angles | 0.17 | 0.21 | 0.28 | 0.05 | 0.13 | 0.00 | 0.08 | 0.04 | 0.02 | 0.02 | 0.00 | 0.00 | <0.1 |
| Cis prolines | 0.00 | 0.00 | 0.00 | 6.38 | 0.00 | 0.00 | 2.27 | 0.00 | 0.00 | 0.00 | 0.00 | 0.00 | <5 |
| CaBLAM outliers | 3.60 | 5.58 | 5.29 | 4.61 | - | 7.85 | 4.50 | 0.12 | 1.99 | 1.19 | - | - | <1.0 |
| CA geometry outliers | 0.00 | 2.09 | 0.71 | 0.89 | - | 1.09 | 0.90 | 0.11 | 0.36 | 0.00 | - | - | <0.5 |

This table is similar to Table 1 but is more inclusive.

^1, 2^ All score values are percent (%) except Clash score and *Molprobity* score, which are percentile, as defined below.

^3^ Clash score is the number of serious steric overlaps (>0.4 Å) per 1000 atoms.

^4^ *Molprobity* score combines clash, rotamer, and Ramachandran evaluations into a single score, normalized to be on the same scale as X-ray resolution. For both Clash score and *Molprobity* score the values are percentile (100^th^ is best, 0^th^ is worst) relative to a set of comparable structures determined for each calculation (see *Molprobity* server for details).

Colors compare score versus goal; green (best), yellow (intermediate), orange (worst). Adapted from *Molprobity* website.
