# Supplementary material for: Expression of quasi-equivalence and capsid dimorphism in the Hepadnaviridae
Source: PLoS Comput Biol. 2020 Apr 20;16(4):e1007782. doi: 10.1371/journal.pcbi.1007782 (PMC7192502; doi:10.1371/journal.pcbi.1007782)
Supplement: S3 Table — 1 All structures are T = 4 capsids except 3KXS, which is a core-antigen dimer complex. All structures are apo, i.e. non-liganded. (DOCX) [file pcbi.1007782.s007.docx]

**S3 Table. All-atom RMSD (Å) of chain-pairs in select core-antigen structures.**^1^

|  | 3KXS | 1QGT | 2G33 | 3J2V | This study |
| --- | --- | --- | --- | --- | --- |
| AB | 1.77 | 0.86 | 1.25 | 1.19 | 0.88 |
| AC | 1.47 | 0.97 | 2.45 | 1.04 | 1.05 |
| AD | 1.13 | 0.54 | 1.50 | 0.79 | 0.58 |
| BC | 1.31 | 1.25 | 2.28 | 2.18 | 1.33 |
| BD | 1.58 | 1.04 | 1.88 | 1.26 | 0.90 |
| CD | 1.59 | 1.04 | 2.30 | 0.89 | 1.05 |

^1^ All structures are T=4 capsids except 3KXS, which is a core-antigen dimer complex. All structures are apo, i.e. non-liganded.
