# Supplementary material for: Expression of quasi-equivalence and capsid dimorphism in the Hepadnaviridae
Source: PLoS Comput Biol. 2020 Apr 20;16(4):e1007782. doi: 10.1371/journal.pcbi.1007782 (PMC7192502; doi:10.1371/journal.pcbi.1007782)
Supplement: S4 Table — 1 All the chain pairs in the table were analyzed with DynDom. However, when the chain pairs did not meet the specific requirements of being “dynamic”, even if their conformations were different, then no values were returned by the program and the cells were left blank. 2 Indicates that chains from T = 3 capsids were compared to chains from T = 4 capsids, in the order given. (DOCX) [file pcbi.1007782.s008.docx]

**S4 Table. Conformational analysis of chain pairs with *DynDom*.**^1^

|  | RMSD | D1 RMSD | D2 RMSD | D1 Size | D2 Size | D1 Residues | D2 Residues | Rotation angle (°) | Translation (Å) | Closure (%) | Hinge Residues |
| --- | --- | --- | --- | --- | --- | --- | --- | --- | --- | --- | --- |
| 1QGT |  |  |  |  |  |  |  |  |  |  |  |
| T=4 AB | 0.86 | 0.44 | 0.82 | 109 | 29 | 3-15, 24-118, 138-138 | 16-23, 119-137, 139-140 | 10.2 | -0.2 | 95.1 | 14-17, 22-24, 118-119, 137-139 |
| T=4 AC | 0.97 | 0.42 | 0.39 | 90 | 22 | 201-109 | 7-19, 110-118 | 4.6 | -0.2 | 99.1 | 19-24, 109-110 |
| T=4 AD | 0.54 | 0.31 | 0.41 | 106 | 28 | 5-8, 18-118, 138-138 | 9-17, 119-137 | 6.8 | 0.1 | 60.2 | 8-9, 17-18, 118-119, 135-138 |
| T=4 BC | 1.25 | 0.62 | 1.29 | 116 | 22 | 3-5, 19-119, 139-140 | 16-18, 120-138 | 15.4 | 0.7 | 52.5 | 15-16, 18-19, 119-120, 138-140 |
| T=4 BD | 1.04 | 0.44 | 0.73 | 107 | 25 | 3-15, 24-117 | 16-19,118-138 | 7.0 | -0.5 | 68.4 | 12-17, 19-24, 117-118 |
| T=4 CD | 1.04 |  |  |  |  |  |  |  |  |  |  |
|  |  |  |  |  |  |  |  |  |  |  |  |
| 2G33 |  |  |  |  |  |  |  |  |  |  |  |
| T=4 AB | 1.25 |  |  |  |  |  |  |  |  |  |  |
| T=4 AC | 2.45 | 0.94 | 1.03 | 114 | 22 | 5-118 | 119-140 | 15.9 | 0.4 | 37.2 | 118-119 |
| T=4 AD | 1.50 |  |  |  |  |  |  |  |  |  |  |
| T=4 BC | 2.28 | 0.91 | 0.96 | 115 | 20 | 5-118, 139-139 | 119-138 | 21.0 | 0.9 | 40.1 | 118-119, 137-139 |
| T=4 BD | 1.88 |  |  |  |  |  |  |  |  |  |  |
| T=4 CD | 2.30 |  |  |  |  |  |  |  |  |  |  |
|  |  |  |  |  |  |  |  |  |  |  |  |
| 3J2V |  |  |  |  |  |  |  |  |  |  |  |
| T=4 AB | 1.19 |  |  |  |  |  |  |  |  |  |  |
| T=4 AC | 1.04 |  |  |  |  |  |  |  |  |  |  |
| T=4 AD | 0.79 | 0.66 | 0.25 | 111 | 20 | 7-117 | 118-137 | 8.9 | 0.0 | 55.6 | 117-118 |
| T=4 BC | 2.18 |  |  |  |  |  |  |  |  |  |  |
| T=4 BD | 1.26 | 0.76 | 0.52 | 69 | 63 | 7-20,33-54, 106-138 | 21-32, 55-105 | 6.2 | 0.3 | 48.5 | 20-25, 32-33, 54-55, 103-106 |
| T=4 CD | 0.89 |  |  |  |  |  |  |  |  |  |  |
|  |  |  |  |  |  |  |  |  |  |  |  |
| 3KXS |  |  |  |  |  |  |  |  |  |  |  |
| T=4 AB | 1.77 |  |  |  |  |  |  |  |  |  |  |
| T=4 AC | 1.47 | 0.42 | 1.08 | 89 | 31 | 4-63, 95-123 | 64-94 | 9.7 | 1.2 | 91.9 | 63-64, 94-95 |
| T=4 AD | 1.13 |  |  |  |  |  |  |  |  |  |  |
| T=4 BC | 1.31 | 0.42 | 0.64 | 91 | 20 | 5-73, 96-117 | 118-137 | 14.5 | -0.9 | 83.9 | 114-118 |
| T=4 BD | 1.58 | 0.81 | 1.65 | 110 | 21 | 6-74, 96-136 | 75-95 | 25.4 | 1.0 | 30.1 | 65-75, 95-96 |
| T=4 CD | 1.59 | 1.09 | 0.75 | 104 | 33 | 3-14, 19-110 | 15-18, 111-139 | 12.7 | 0.3 | 100.0 | 14-15, 18-19, 110-111 |
|  |  |  |  |  |  |  |  |  |  |  |  |
| 4G93 |  |  |  |  |  |  |  |  |  |  |  |
| T=4 AB | 1.24 |  |  |  |  |  |  |  |  |  |  |
| T=4 AC | 1.75 |  |  |  |  |  |  |  |  |  |  |
| T=4 AD | 1.51 |  |  |  |  |  |  |  |  |  |  |
| T=4 BC | 1.79 | 1.07 | 0.85 | 76 | 24 | 5-64, 96-111 | 112-135 | 18.6 | 0.4 | 14.3 | 111-113 |
| T=4 BD | 1.74 | 0.94 | 1.03 | 84 | 22 | 7-93, 97-113 | 114-135 | 12.9 | 0.4 | 2.7 | 113-115 |
| T=4 CD | 1.31 |  |  |  |  |  |  |  |  |  |  |
|  |  |  |  |  |  |  |  |  |  |  |  |
| 6BVF |  |  |  |  |  |  |  |  |  |  |  |
| T=4 AB | 1.20 |  |  |  |  |  |  |  |  |  |  |
| T=4 AC | 1.71 |  |  |  |  |  |  |  |  |  |  |
| T=4 AD | 1.21 |  |  |  |  |  |  |  |  |  |  |
| T=4 BC | 1.56 |  |  |  |  |  |  |  |  |  |  |
| T=4 BD | 1.25 |  |  |  |  |  |  |  |  |  |  |
| T=4 CD | 1.38 |  |  |  |  |  |  |  |  |  |  |
|  |  |  |  |  |  |  |  |  |  |  |  |
| 6BVN |  |  |  |  |  |  |  |  |  |  |  |
| T=3 AB | 0.90 |  |  |  |  |  |  |  |  |  |  |
| T=3 AC | 1.34 |  |  |  |  |  |  |  |  |  |  |
| T=3 BC | 1.59 |  |  |  |  |  |  |  |  |  |  |
|  |  |  |  |  |  |  |  |  |  |  |  |
| 6BVN chains compared to 6BVF chains^2^ | | | | | | | | | | | |
| AA | 1.07 |  |  |  |  |  |  |  |  |  |  |
| AB | 0.91 |  |  |  |  |  |  |  |  |  |  |
| AC | 1.58 |  |  |  |  |  |  |  |  |  |  |
| AD | 1.05 |  |  |  |  |  |  |  |  |  |  |
| BA | 1.15 |  |  |  |  |  |  |  |  |  |  |
| BB | 0.81 |  |  |  |  |  |  |  |  |  |  |
| BC | 1.71 |  |  |  |  |  |  |  |  |  |  |
| BD | 1.24 |  |  |  |  |  |  |  |  |  |  |
| CA | 1.37 |  |  |  |  |  |  |  |  |  |  |
| CB | 1.39 |  |  |  |  |  |  |  |  |  |  |
| CC | 1.63 |  |  |  |  |  |  |  |  |  |  |
| CD | 1.07 |  |  |  |  |  |  |  |  |  |  |
|  |  |  |  |  |  |  |  |  |  |  |  |
| This study | | | | | | | | | | | |
| T=4 AB | 0.88 | 0.53 | 0.33 | 80 | 33 | 4-12, 21-39, 63-114 | 13-20, 115-139 | 9.0 | -0.2 | 93.9 | 12-13, 20-25, 114-115 |
| T=4 AC | 1.05 | 0.50 | 1.35 | 116 | 22 | 3-13, 15-16, 19-121 | 14-14, 17-28, 122-140 | 12.5 | 0.3 | 37.1 | 13-20, 121-122 |
| T=4 AD | 0.58 |  |  |  |  |  |  |  |  |  |  |
| T=4 BC | 1.33 |  |  |  |  |  |  |  |  |  |  |
| T=4 BD | 0.90 |  |  |  |  |  |  |  |  |  |  |
| T=4 CD | 1.05 |  |  |  |  |  |  |  |  |  |  |
|  |  |  |  |  |  |  |  |  |  |  |  |
| T=3 AB | 0.85 |  |  |  |  |  |  |  |  |  |  |
| T=3 AC | 1.09 | 0.76 | 0.76 | 114 | 22 | 4-14, 16-118 | 15-15, 119-139 | 14.6 | 0.1 | 28.2 | 14-16, 118-119 |
| T=3 BC | 1.27 |  |  |  |  |  |  |  |  |  |  |
|  |  |  |  |  |  |  |  |  |  |  |  |
| T=3 chains compared to T=4 chains^2^ | | | | | | | | | | | |
| AA | 0.57 |  |  |  |  |  |  |  |  |  |  |
| AB | 0.93 |  |  |  |  |  |  |  |  |  |  |
| AC | 1.06 | 0.60 | 1.21 | 109 | 25 | 5-10, 16-118 | 11-15, 119-138 | 11.4 | -0.1 | 18.1 | 8-11,15-16, 118-119 |
| AD | 0.69 |  |  |  |  |  |  |  |  |  |  |
| BA | 0.95 |  |  |  |  |  |  |  |  |  |  |
| BB | 0.74 |  |  |  |  |  |  |  |  |  |  |
| BC | 1.29 |  |  |  |  |  |  |  |  |  |  |
| BD | 0.98 |  |  |  |  |  |  |  |  |  |  |
| CA | 1.13 | 0.69 | 1.24 | 118 | 20 | 3-16, 20-123 | 17-19, 124-140 | 13.6 | 0.2 | 43.3 | 15-20, 123-124 |
| CB | 1.38 | 0.74 | 0.78 | 118 | 20 | 3-118, 139-140 | 119-138 | 18.9 | 0.5 | 43.5 | 118-119, 138-139 |
| CC | 0.65 |  |  |  |  |  |  |  |  |  |  |
| CD | 1.11 |  |  |  |  |  |  |  |  |  |  |

^1^ All the chain pairs in the table were analyzed with *DynDom*. However, when the chain pairs did not meet the specific requirements of being “dynamic”, even if their conformations were different, then no values were returned by the program and the cells were left blank.

^2^ Indicates that chains from T=3 capsids were compared to chains from T=4 capsids, in the order given.
