# Supplementary material for: Expression of quasi-equivalence and capsid dimorphism in the Hepadnaviridae
Source: PLoS Comput Biol. 2020 Apr 20;16(4):e1007782. doi: 10.1371/journal.pcbi.1007782 (PMC7192502; doi:10.1371/journal.pcbi.1007782)
Supplement: S6 Table — 1 Results from a KFC2 computational alanine scanning interface analysis of the three quasi-equivalent sites (AA, BC, and CB) in the T = 3 capsids described in this study. Residues identified as hot spots by KFC2 are listed separately for each chain in a dimer. Residues in bold font were classed as high confidence by KFC2. Area and volume values are for the interface region between the two chains, 3 Å from each chain, as calculated with FADE (on the KFC2 server) and UCSF Chimera. Note that the CB interface is smaller and that V124 and R127 are not classed as part of the CB interface (boxed cells). (DOCX) [file pcbi.1007782.s010.docx]

**S6 Table. Computational alanine scanning interface analysis of T=3 capsids.**^1^

|  | **AA** | | **BC** | | **CB** | |
| --- | --- | --- | --- | --- | --- | --- |
|  |  |  |  |  | **F18** |  |
|  |  | **F23** |  | **F23** |  | **F23** |
|  |  | P25 |  | P25 |  |  |
|  |  |  |  | **L30** |  |  |
|  |  |  |  | A36 |  |  |
|  |  |  | V120 |  |  |  |
|  |  | **F122** |  | **F122** |  | **F122** |
|  | V124 |  | V124 |  |  |  |
|  | R127 |  | **R127** |  |  |  |
|  |  |  | T128 |  |  |  |
|  | Y132 |  | Y132 |  | Y132 |  |
|  | P134 |  |  |  |  |  |
|  |  | I139 |  |  |  |  |
|  |  |  |  | |  | |
| Area (Å^2^) | 1915 | | 1879 | | 1660 | |
| Vol (Å^3^) | 2326 | | 2260 | | 1424 | |

^1^ Results from a KFC2 computational alanine scanning interface analysis of the three quasi-equivalent sites (AA, BC, and CB) in the T=3 capsids described in this study. Residues identified as hot spots by KFC2 are listed separately for each chain in a dimer. Residues in bold font were classed as high confidence by KFC2. Area and volume values are for the interface region between the two chains, 3 Å from each chain, as calculated with FADE (on the KFC2 server) and Chimera. Note that the CB interface is smaller and that V124 and R127 are not classed as part of the CB interface (boxed cells).
