# Supplementary material for: Expression of quasi-equivalence and capsid dimorphism in the Hepadnaviridae
Source: PLoS Comput Biol. 2020 Apr 20;16(4):e1007782. doi: 10.1371/journal.pcbi.1007782 (PMC7192502; doi:10.1371/journal.pcbi.1007782)
Supplement: S7 Table — (DOCX) [file pcbi.1007782.s011.docx]

**S7 Table. *PISA* intra-dimer interface analysis of T=3 and T=4 capsids.**

| Dimer | Residues | Atoms | Area (Å^2^) | N_HB_ | N_SB_ | ΔG (kCal/mol) |
| --- | --- | --- | --- | --- | --- | --- |
| T=3 AB | 50/47 | 196/188 | 1973.1 | 6 | 17 | -37.0 |
| T=3 CC | 49/49 | 197/197 | 2002.7 | 6 | 14 | -30.6 |
| T=4 AB | 50/46 | 197/193 | 1964.4 | 3 | 9 | -33.6 |
| T=4 CD | 46/48 | 191/194 | 1934.6 | 3 | 6 | -40.3 |
