# Supplementary material for: Expression of quasi-equivalence and capsid dimorphism in the Hepadnaviridae
Source: PLoS Comput Biol. 2020 Apr 20;16(4):e1007782. doi: 10.1371/journal.pcbi.1007782 (PMC7192502; doi:10.1371/journal.pcbi.1007782)
Supplement: S1 Appendix — (PDF) [file pcbi.1007782.s012.pdf]

Full wwPDB/EMDataBank EM Map/Model Validation Report (i)

Oct 28, 2019 – 04:12 PM EDT

PDB ID	:	6UI6
EMDB ID:	:	EMD-20669
Title	:	HBV T=3 149C3A
Authors	:	Wu, W.; Watts, N.R.; Cheng, N.; Huang, R.; Steven, A.; Wingfield, P.T.
Deposited on	:	2019-09-30
Resolution	:	3.53 Å(reported)
Based on PDB ID	:	1QGT

This is a Full wwPDB/EMDataBank EM Map/Model Validation Report for a publicly released PDB/EMDB entry.

We welcome your comments at *validation@mail.wwpdb.org*
A user guide is available at
https://www.wwpdb.org/validation/2017/EMValidationReportHelp
with specific help available everywhere you see the (i) symbol.

MolProbity	:	4.02b-467
Percentile statistics	:	20171227.v01 (using entries in the PDB archive December 27th 2017)
Ideal geometry (proteins)	:	Engh & Huber (2001)
Ideal geometry (DNA, RNA)	:	Parkinson et. al. (1996)
Validation Pipeline (wwPDB-VP)	:	2.4

1 Overall quality at a glance (i)

The following experimental techniques were used to determine the structure:
ELECTRON MICROSCOPY

The reported resolution of this entry is 3.53 Å.

Percentile scores (ranging between 0-100) for global validation metrics of the entry are shown in the following graphic. The table shows the number of entries on which the scores are based.

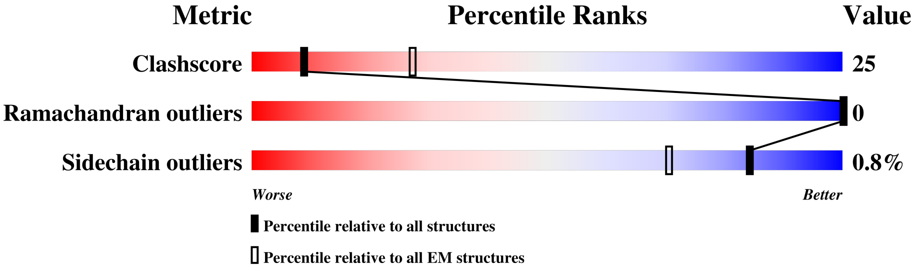

Metric	Whole archive (#Entries)	EM structures (#Entries)
Clashscore	136327	1886
Ramachandran outliers	132723	1663
Sidechain outliers	132532	1531

The table below summarises the geometric issues observed across the polymeric chains. The red, orange, yellow and green segments on the bar indicate the fraction of residues that contain outliers for >=3, 2, 1 and 0 types of geometric quality criteria. A grey segment represents the fraction of residues that are not modelled. The numeric value for each fraction is indicated below the corresponding segment, with a dot representing fractions <=5%

Mol	Chain	Length	Quality of chain
1	A	143	76% 22% ••
1	B	143	71% 29%
1	C	143	81% 18% •

2 Entry composition (i)

There is only 1 type of molecule in this entry. The entry contains 6742 atoms, of which 3335 are hydrogens and 0 are deuteriums.

In the tables below, the AltConf column contains the number of residues with at least one atom in alternate conformation and the Trace column contains the number of residues modelled with at most 2 atoms.

- Molecule 1 is a protein called Core protein.

Mol	Chain	Residues	Atoms						AltConf	Trace
			Total	C	H	N	O	S		
1	A	142	2241	735	1108	188	207	3	0	0
1	B	143	2260	741	1119	189	208	3	0	0
1	C	142	2241	735	1108	188	207	3	0	0

There are 9 discrepancies between the modelled and reference sequences:

Chain	Residue	Modelled	Actual	Comment	Reference
A	48	ALA	CYS	conflict	UNP A0A0D4D613
A	61	ALA	CYS	conflict	UNP A0A0D4D613
A	107	ALA	CYS	conflict	UNP A0A0D4D613
B	48	ALA	CYS	conflict	UNP A0A0D4D613
B	61	ALA	CYS	conflict	UNP A0A0D4D613
B	107	ALA	CYS	conflict	UNP A0A0D4D613
C	48	ALA	CYS	conflict	UNP A0A0D4D613
C	61	ALA	CYS	conflict	UNP A0A0D4D613
C	107	ALA	CYS	conflict	UNP A0A0D4D613

3 Residue-property plots (i)

These plots are drawn for all protein, RNA and DNA chains in the entry. The first graphic for a chain summarises the proportions of the various outlier classes displayed in the second graphic. The second graphic shows the sequence view annotated by issues in geometry. Residues are color-coded according to the number of geometric quality criteria for which they contain at least one outlier: green = 0, yellow = 1, orange = 2 and red = 3 or more. Stretches of 2 or more consecutive residues without any outlier are shown as a green connector. Residues present in the sample, but not in the model, are shown in grey.

- Molecule 1: Core protein

Chain A:

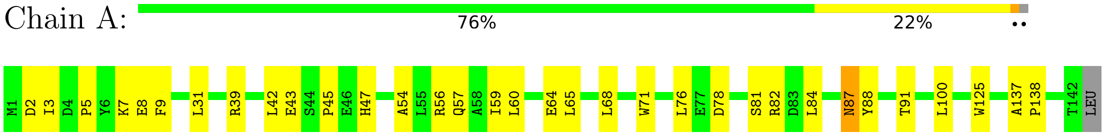

- Molecule 1: Core protein

Chain B:

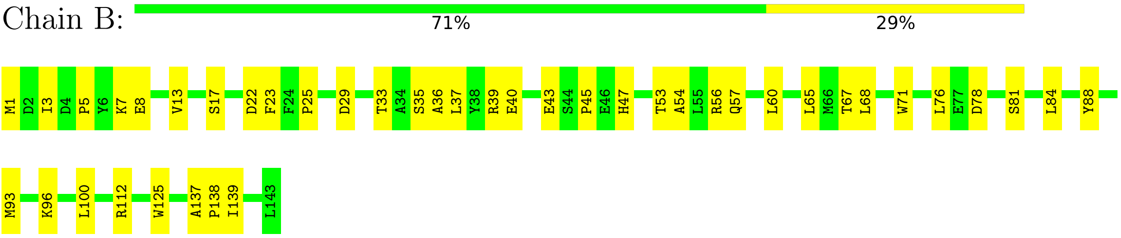

- Molecule 1: Core protein

Chain C:

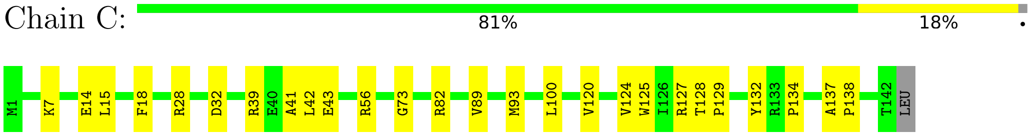

4 Experimental information (i)

Property	Value	Source
Reconstruction method	SINGLE PARTICLE	Depositor
Imposed symmetry	POINT, Not provided	Depositor
Number of particles used	15000	Depositor
Resolution determination method	FSC 0.143 CUT-OFF	Depositor
CTF correction method	PHASE FLIPPING AND AMPLITUDE CORRECTION	Depositor
Microscope	FEI POLARA 300	Depositor
Voltage (kV)	300	Depositor
Electron dose $(e^-/\text{Å}^2)$	25	Depositor
Minimum defocus (nm)	Not provided	Depositor
Maximum defocus (nm)	Not provided	Depositor
Magnification	Not provided	Depositor
Image detector	GATAN K2 SUMMIT (4k x 4k)	Depositor

5 Model quality (i)

5.1 Standard geometry (i)

The Z score for a bond length (or angle) is the number of standard deviations the observed value is removed from the expected value. A bond length (or angle) with $|Z| > 5$ is considered an outlier worth inspection. RMSZ is the root-mean-square of all Z scores of the bond lengths (or angles).

Mol	Chain	Bond lengths		Bond angles	
		RMSZ	#|Z| >2	RMSZ	#|Z| >2
1	A	0.51	0/1167	0.59	1/1599 (0.1%)
1	B	0.49	0/1175	0.59	0/1610
1	C	0.45	0/1167	0.57	1/1599 (0.1%)
All	All	0.48	0/3509	0.58	2/4808 (0.0%)

There are no bond length outliers.

All (2) bond angle outliers are listed below:

Mol	Chain	Res	Type	Atoms	Z	Observed(°)	Ideal(°)
1	C	100	LEU	CB-CG-CD2	7.18	123.20	111.00
1	A	82	ARG	NE-CZ-NH1	5.06	122.83	120.30

There are no chirality outliers.

There are no planarity outliers.

5.2 Too-close contacts (i)

In the following table, the Non-H and H(model) columns list the number of non-hydrogen atoms and hydrogen atoms in the chain respectively. The H(added) column lists the number of hydrogen atoms added and optimized by MolProbity. The Clashes column lists the number of clashes within the asymmetric unit, whereas Symm-Clashes lists symmetry related clashes.

Mol	Chain	Non-H	H(model)	H(added)	Clashes	Symm-Clashes
1	A	1133	1108	1116	126	0
1	B	1141	1119	1126	154	0
1	C	1133	1108	1116	41	0
All	All	3407	3335	3358	171	0

The all-atom clashscore is defined as the number of clashes found per 1000 atoms (including hydrogen atoms). The all-atom clashscore for this structure is 25.

All (171) close contacts within the same asymmetric unit are listed below, sorted by their clash

magnitude.

Atom-1	Atom-2	Interatomic distance (Å)	Clash overlap (Å)
1:A:47:HIS:CE1	1:B:8:GLU:HG3	1.49	1.47
1:A:88:TYR:CD2	1:B:68:LEU:CD1	2.13	1.31
1:A:68:LEU:CD1	1:B:88:TYR:CD2	2.13	1.30
1:A:47:HIS:CE1	1:B:8:GLU:CG	2.12	1.29
1:A:64:GLU:OE1	1:B:96:LYS:NZ	1.69	1.25
1:A:88:TYR:CD2	1:B:68:LEU:HD13	1.75	1.20
1:A:47:HIS:ND1	1:B:8:GLU:CD	1.96	1.17
1:A:68:LEU:HD13	1:B:88:TYR:CD2	1.79	1.14
1:A:47:HIS:CE1	1:B:8:GLU:CD	2.23	1.11
1:A:5:PRO:HB3	1:B:60:LEU:HD11	1.33	1.07
1:A:5:PRO:CB	1:B:60:LEU:HD11	1.85	1.05
1:A:68:LEU:CD1	1:B:88:TYR:CE2	2.40	1.04
1:A:84:LEU:HD23	1:B:76:LEU:CD2	1.88	1.04
1:A:68:LEU:HD12	1:B:88:TYR:CD2	1.93	1.03
1:A:88:TYR:CE2	1:B:68:LEU:CD1	2.42	1.01
1:A:88:TYR:CD2	1:B:68:LEU:HD12	1.90	1.01
1:A:68:LEU:HD13	1:B:88:TYR:CE2	1.97	0.98
1:A:68:LEU:CD1	1:B:88:TYR:HD2	1.64	0.98
1:B:37:LEU:CD2	1:C:120:VAL:HG22	1.94	0.97
1:A:88:TYR:CE2	1:B:68:LEU:HD13	2.00	0.95
1:A:8:GLU:OE2	1:B:53:THR:OG1	1.83	0.95
1:B:22:ASP:O	1:C:129:PRO:HG2	1.66	0.95
1:B:25:PRO:HB3	1:C:127:ARG:O	1.67	0.94
1:A:84:LEU:HD23	1:B:76:LEU:HD22	1.46	0.94
1:A:68:LEU:HD12	1:B:88:TYR:CE2	2.03	0.93
1:A:47:HIS:HD1	1:B:8:GLU:CD	1.72	0.92
1:A:64:GLU:CD	1:B:96:LYS:NZ	2.24	0.91
1:B:33:THR:HA	1:C:18:PHE:CE1	2.06	0.90
1:A:88:TYR:CE2	1:B:68:LEU:HD12	2.06	0.90
1:A:64:GLU:CD	1:B:96:LYS:CD	2.42	0.88
1:A:88:TYR:HD2	1:B:68:LEU:CD1	1.78	0.87
1:A:88:TYR:HD2	1:B:68:LEU:HD13	1.27	0.87
1:B:29:ASP:HB3	1:C:127:ARG:CG	2.06	0.86
1:A:47:HIS:HE1	1:B:8:GLU:HG3	1.37	0.86
1:A:64:GLU:OE1	1:B:96:LYS:CD	2.25	0.85
1:B:40:GLU:OE2	1:C:7:LYS:HE3	1.76	0.85
1:B:37:LEU:HD22	1:C:120:VAL:HG22	1.60	0.84
1:A:5:PRO:HB3	1:B:60:LEU:CD1	2.07	0.84
1:A:64:GLU:OE1	1:B:96:LYS:CE	2.28	0.81
1:A:76:LEU:CD2	1:B:84:LEU:HD23	2.12	0.80
1:A:84:LEU:CD2	1:B:76:LEU:HD22	2.11	0.80

Continued on next page...

Continued from previous page...

Atom-1	Atom-2	Interatomic distance (Å)	Clash overlap (Å)
1:A:68:LEU:HD13	1:B:88:TYR:HD2	1.29	0.79
1:A:88:TYR:CD1	1:B:71:TRP:CE3	2.71	0.79
1:A:64:GLU:CD	1:B:96:LYS:HD3	2.03	0.79
1:A:78:ASP:HB2	1:B:78:ASP:OD2	1.82	0.79
1:B:29:ASP:HB3	1:C:127:ARG:HG2	1.64	0.78
1:A:42:LEU:HB3	1:B:3:ILE:HD12	1.63	0.78
1:A:100:LEU:CD1	1:B:57:GLN:OE1	2.31	0.78
1:A:100:LEU:HD11	1:B:57:GLN:OE1	1.85	0.77
1:A:42:LEU:HB3	1:B:3:ILE:CD1	2.16	0.76
1:B:23:PHE:O	1:C:129:PRO:CD	2.34	0.75
1:A:64:GLU:OE1	1:B:96:LYS:HD2	1.86	0.74
1:A:84:LEU:CD2	1:B:76:LEU:CD2	2.65	0.74
1:C:39:ARG:NH2	1:C:43:GLU:OE2	2.20	0.74
1:A:7:LYS:HG2	1:B:43:GLU:O	1.87	0.73
1:B:22:ASP:O	1:C:129:PRO:CG	2.36	0.73
1:A:5:PRO:HB2	1:B:60:LEU:HD11	1.71	0.72
1:C:41:ALA:O	1:C:56:ARG:NH2	2.23	0.72
1:C:42:LEU:O	1:C:56:ARG:NH1	2.24	0.71
1:A:47:HIS:NE2	1:B:8:GLU:HG3	2.04	0.70
1:A:84:LEU:HD23	1:B:76:LEU:HD21	1.71	0.70
1:B:36:ALA:HB1	1:C:15:LEU:HA	1.73	0.70
1:A:47:HIS:ND1	1:B:8:GLU:OE1	2.24	0.70
1:B:37:LEU:HD21	1:C:120:VAL:HG22	1.72	0.69
1:B:29:ASP:HB3	1:C:127:ARG:HG3	1.74	0.69
1:A:64:GLU:OE2	1:B:96:LYS:NZ	2.25	0.69
1:A:39:ARG:NH2	1:A:43:GLU:OE2	2.27	0.68
1:A:76:LEU:HD21	1:B:84:LEU:HD23	1.76	0.68
1:A:47:HIS:CE1	1:B:8:GLU:OE2	2.47	0.67
1:A:43:GLU:OE2	1:B:3:ILE:HA	1.95	0.66
1:A:76:LEU:HD23	1:B:84:LEU:HD23	1.78	0.66
1:A:7:LYS:HE3	1:B:45:PRO:HD3	1.77	0.66
1:A:8:GLU:HB2	1:B:47:HIS:CD2	2.32	0.65
1:C:124:VAL:O	1:C:128:THR:OG1	2.07	0.64
1:A:5:PRO:HB3	1:B:60:LEU:HD21	1.79	0.64
1:A:57:GLN:OE1	1:B:100:LEU:HD11	1.98	0.63
1:A:57:GLN:HG3	1:B:54:ALA:HB1	1.80	0.63
1:A:8:GLU:HB2	1:B:47:HIS:CG	2.34	0.63
1:B:33:THR:HA	1:C:18:PHE:CZ	2.32	0.63
1:A:8:GLU:CD	1:B:56:ARG:HH12	2.01	0.63
1:A:47:HIS:ND1	1:B:8:GLU:OE2	2.32	0.63
1:A:65:LEU:HD21	1:B:93:MET:HE2	1.82	0.62

Continued on next page...

Continued from previous page...

Atom-1	Atom-2	Interatomic distance (Å)	Clash overlap (Å)
1:A:88:TYR:HB2	1:B:71:TRP:CZ3	2.35	0.61
1:B:35:SER:OG	1:C:14:GLU:OE2	2.11	0.61
1:A:64:GLU:HB3	1:B:93:MET:SD	2.39	0.60
1:A:2:ASP:O	1:B:43:GLU:OE2	2.18	0.60
1:B:33:THR:CA	1:C:18:PHE:CE1	2.82	0.60
1:A:87:ASN:ND2	1:A:91:THR:OG1	2.34	0.60
1:B:39:ARG:NH2	1:B:43:GLU:OE2	2.33	0.60
1:A:81:SER:HA	1:B:76:LEU:CD1	2.32	0.59
1:C:125:TRP:CZ2	1:C:137:ALA:HB2	2.38	0.59
1:A:8:GLU:OE2	1:B:53:THR:CB	2.50	0.59
1:A:56:ARG:NH1	1:B:8:GLU:OE1	2.36	0.58
1:A:64:GLU:CD	1:B:96:LYS:HZ2	1.87	0.57
1:A:71:TRP:CZ3	1:B:88:TYR:HB2	2.39	0.57
1:A:100:LEU:HD13	1:B:57:GLN:OE1	2.04	0.57
1:B:23:PHE:O	1:C:129:PRO:HD3	2.05	0.56
1:B:139:ILE:HG21	1:C:134:PRO:HG3	1.88	0.56
1:A:88:TYR:CE2	1:B:68:LEU:HA	2.41	0.56
1:A:57:GLN:OE1	1:B:100:LEU:CD1	2.55	0.55
1:C:28:ARG:NH2	1:C:32:ASP:OD1	2.38	0.55
1:B:13:VAL:O	1:B:17:SER:N	2.39	0.55
1:A:88:TYR:CD1	1:B:71:TRP:CD2	2.93	0.55
1:A:76:LEU:HD23	1:B:84:LEU:CD2	2.37	0.55
1:A:56:ARG:NH1	1:B:8:GLU:CD	2.60	0.54
1:B:33:THR:N	1:C:18:PHE:HE1	2.05	0.54
1:B:25:PRO:HB3	1:C:127:ARG:C	2.28	0.54
1:A:78:ASP:CB	1:B:78:ASP:OD2	2.55	0.53
1:A:64:GLU:CD	1:B:96:LYS:CE	2.75	0.53
1:A:8:GLU:OE2	1:B:53:THR:HG23	2.08	0.53
1:A:8:GLU:CD	1:B:56:ARG:NH1	2.61	0.53
1:A:47:HIS:HE1	1:B:8:GLU:CG	2.04	0.53
1:A:68:LEU:CD1	1:B:88:TYR:HE2	2.18	0.52
1:B:137:ALA:HB1	1:B:138:PRO:HD2	1.92	0.51
1:A:5:PRO:HB3	1:B:60:LEU:CD2	2.40	0.51
1:A:71:TRP:CE3	1:B:88:TYR:CD1	2.99	0.51
1:B:139:ILE:CG2	1:C:134:PRO:HG3	2.40	0.51
1:B:25:PRO:CB	1:C:127:ARG:O	2.51	0.51
1:A:5:PRO:CB	1:B:60:LEU:CD1	2.73	0.51
1:A:84:LEU:CD2	1:B:76:LEU:HD21	2.38	0.50
1:B:33:THR:CA	1:C:18:PHE:HE1	2.23	0.50
1:A:81:SER:HA	1:B:76:LEU:HD11	1.92	0.50
1:C:125:TRP:CH2	1:C:137:ALA:HB2	2.47	0.50

Continued on next page...

Continued from previous page...

Atom-1	Atom-2	Interatomic distance (Å)	Clash overlap (Å)
1:C:137:ALA:HB1	1:C:138:PRO:HD2	1.93	0.50
1:A:45:PRO:HA	1:B:7:LYS:O	2.12	0.49
1:A:68:LEU:HA	1:B:88:TYR:CE2	2.47	0.49
1:B:23:PHE:CD1	1:C:132:TYR:HB2	2.46	0.49
1:A:76:LEU:HD22	1:B:81:SER:HA	1.95	0.49
1:A:78:ASP:OD2	1:B:78:ASP:HB2	2.11	0.49
1:A:8:GLU:CD	1:B:53:THR:HG1	2.13	0.49
1:A:76:LEU:CD2	1:B:81:SER:HA	2.42	0.49
1:A:8:GLU:OE2	1:B:53:THR:CG2	2.60	0.49
1:A:9:PHE:CE2	1:B:47:HIS:CE1	3.01	0.48
1:B:23:PHE:HD1	1:C:132:TYR:HB2	1.78	0.48
1:B:125:TRP:CZ2	1:B:137:ALA:HB2	2.47	0.48
1:B:139:ILE:HD12	1:C:134:PRO:CD	2.43	0.48
1:A:43:GLU:O	1:B:7:LYS:HG2	2.14	0.48
1:A:137:ALA:HB1	1:A:138:PRO:HD2	1.95	0.48
1:A:65:LEU:CD1	1:B:65:LEU:HD21	2.44	0.47
1:A:88:TYR:OH	1:B:67:THR:HG22	2.15	0.47
1:A:47:HIS:HA	1:B:8:GLU:OE1	2.13	0.47
1:A:125:TRP:CE2	1:A:137:ALA:HB2	2.50	0.47
1:A:68:LEU:HD11	1:B:88:TYR:HD2	1.67	0.46
1:B:23:PHE:HD1	1:C:132:TYR:CB	2.28	0.46
1:A:68:LEU:HD13	1:B:88:TYR:HE2	1.68	0.46
1:A:88:TYR:CD1	1:B:71:TRP:CZ3	3.02	0.46
1:B:23:PHE:CD1	1:C:132:TYR:CB	2.99	0.46
1:A:31:LEU:CD2	1:A:59:ILE:HD12	2.45	0.46
1:A:42:LEU:HB3	1:B:3:ILE:HD11	1.95	0.45
1:A:65:LEU:CD2	1:B:93:MET:CE	2.94	0.45
1:A:65:LEU:HD21	1:B:93:MET:CE	2.45	0.45
1:B:23:PHE:O	1:C:129:PRO:HD2	2.15	0.45
1:A:88:TYR:CE1	1:B:71:TRP:CD2	3.05	0.44
1:A:2:ASP:H	1:B:39:ARG:HH12	1.64	0.44
1:A:64:GLU:OE2	1:B:96:LYS:HD3	2.16	0.44
1:C:89:VAL:HG22	1:C:93:MET:CE	2.48	0.44
1:A:2:ASP:H	1:B:39:ARG:NH1	2.16	0.44
1:A:9:PHE:CE2	1:B:47:HIS:HE1	2.36	0.43
1:C:73:GLY:HA2	1:C:82:ARG:HE	1.84	0.43
1:A:3:ILE:HA	1:B:43:GLU:OE2	2.20	0.42
1:A:125:TRP:CZ2	1:A:137:ALA:HB2	2.54	0.42
1:A:60:LEU:HD11	1:B:5:PRO:CB	2.50	0.42
1:A:88:TYR:CZ	1:B:68:LEU:HA	2.55	0.41
1:A:65:LEU:HD13	1:B:65:LEU:HD21	2.03	0.41

Continued on next page...

Continued from previous page...

Atom-1	Atom-2	Interatomic distance (Å)	Clash overlap (Å)
1:A:76:LEU:HD13	1:B:81:SER:HB2	2.03	0.41
1:A:78:ASP:O	1:A:81:SER:OG	2.22	0.41
1:A:64:GLU:CD	1:B:96:LYS:HD2	2.28	0.41
1:A:84:LEU:HD23	1:B:76:LEU:CD1	2.50	0.40
1:B:23:PHE:O	1:C:129:PRO:CG	2.69	0.40
1:B:37:LEU:HD22	1:C:120:VAL:CG2	2.41	0.40
1:A:54:ALA:HB1	1:B:57:GLN:HG3	2.02	0.40

There are no symmetry-related clashes.

5.3 Torsion angles (i)

5.3.1 Protein backbone (i)

In the following table, the Percentiles column shows the percent Ramachandran outliers of the chain as a percentile score with respect to all PDB entries followed by that with respect to all EM entries.

The Analysed column shows the number of residues for which the backbone conformation was analysed, and the total number of residues.

Mol	Chain	Analysed	Favoured	Allowed	Outliers	Percentiles	
1	A	140/143 (98%)	135 (96%)	5 (4%)	0	100	100
1	B	141/143 (99%)	133 (94%)	8 (6%)	0	100	100
1	C	140/143 (98%)	133 (95%)	7 (5%)	0	100	100
All	All	421/429 (98%)	401 (95%)	20 (5%)	0	100	100

There are no Ramachandran outliers to report.

5.3.2 Protein sidechains (i)

In the following table, the Percentiles column shows the percent sidechain outliers of the chain as a percentile score with respect to all PDB entries followed by that with respect to all EM entries.

The Analysed column shows the number of residues for which the sidechain conformation was analysed, and the total number of residues.

Mol	Chain	Analysed	Rotameric	Outliers	Percentiles	
1	A	123/124 (99%)	122 (99%)	1 (1%)	83	92
1	B	124/124 (100%)	122 (98%)	2 (2%)	65	85
1	C	123/124 (99%)	123 (100%)	0	100	100
All	All	370/372 (100%)	367 (99%)	3 (1%)	84	92

All (3) residues with a non-rotameric sidechain are listed below:

Mol	Chain	Res	Type
1	A	87	ASN
1	B	1	MET
1	B	112	ARG

Some sidechains can be flipped to improve hydrogen bonding and reduce clashes. All (2) such sidechains are listed below:

Mol	Chain	Res	Type
1	A	52	HIS
1	A	87	ASN

5.3.3 RNA (i)

There are no RNA molecules in this entry.

5.4 Non-standard residues in protein, DNA, RNA chains (i)

There are no non-standard protein/DNA/RNA residues in this entry.

5.5 Carbohydrates (i)

There are no carbohydrates in this entry.

5.6 Ligand geometry (i)

There are no ligands in this entry.

5.7 Other polymers (i)

There are no such residues in this entry.

5.8 Polymer linkage issues (i)

There are no chain breaks in this entry.
