# Supplementary material for: Expression of quasi-equivalence and capsid dimorphism in the Hepadnaviridae
Source: PLoS Comput Biol. 2020 Apr 20;16(4):e1007782. doi: 10.1371/journal.pcbi.1007782 (PMC7192502; doi:10.1371/journal.pcbi.1007782)
Supplement: S2 Appendix — (PDF) [file pcbi.1007782.s013.pdf]

# Full wwPDB/EMDataBank EM Map/Model Validation Report (i)

Oct 28, 2019 – 04:13 PM EDT

| | | |
|---|---|---|
| PDB ID | : | 6UI7 |
| EMDB ID: | : | EMD-20670 |
| Title | : | HBV T=3 149C3A |
| Authors | : | Wu, W.; Watts, N.R.; Cheng, N.; Huang, R.; Steven, A.; Wingfield, P.T. |
| Deposited on | : | 2019-09-30 |
| Resolution | : | 3.65 Å(reported) |
| Based on PDB ID | : | 1QGT |

This is a Full wwPDB/EMDataBank EM Map/Model Validation Report for a publicly released PDB/EMDB entry.

We welcome your comments at *validation@mail.wwpdb.org*
A user guide is available at
https://www.wwpdb.org/validation/2017/EMValidationReportHelp
with specific help available everywhere you see the (i) symbol.

| | | |
|---|---|---|
| MolProbity | : | 4.02b-467 |
| Percentile statistics | : | 20171227.v01 (using entries in the PDB archive December 27th 2017) |
| Ideal geometry (proteins) | : | Engh & Huber (2001) |
| Ideal geometry (DNA, RNA) | : | Parkinson et. al. (1996) |
| Validation Pipeline (wwPDB-VP) | : | 2.4 |

# 1 Overall quality at a glance (i)

The following experimental techniques were used to determine the structure:
*ELECTRON MICROSCOPY*

The reported resolution of this entry is 3.65 Å.

Percentile scores (ranging between 0-100) for global validation metrics of the entry are shown in the following graphic. The table shows the number of entries on which the scores are based.

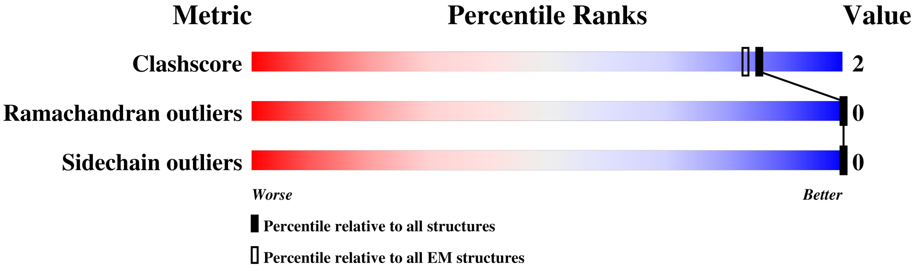


| Metric | Whole archive (#Entries) | EM structures (#Entries) |
|---|---|---|
| Clashscore | 136327 | 1886 |
| Ramachandran outliers | 132723 | 1663 |
| Sidechain outliers | 132532 | 1531 |

The table below summarises the geometric issues observed across the polymeric chains. The red, orange, yellow and green segments on the bar indicate the fraction of residues that contain outliers for >=3, 2, 1 and 0 types of geometric quality criteria. A grey segment represents the fraction of residues that are not modelled. The numeric value for each fraction is indicated below the corresponding segment, with a dot representing fractions <=5%

| Mol | Chain | Length | Quality of chain |
|---|---|---|---|
| 1 | A | 143 | 91% 8% • |
| 1 | B | 143 | 94% 6% |
| 1 | C | 143 | 95% • • |
| 1 | D | 143 | 94% 6% |

# 2 Entry composition (i)

There is only 1 type of molecule in this entry. The entry contains 9034 atoms, of which 4486 are hydrogens and 0 are deuteriums.

In the tables below, the AltConf column contains the number of residues with at least one atom in alternate conformation and the Trace column contains the number of residues modelled with at most 2 atoms.

- Molecule 1 is a protein called Core protein.

| Mol | Chain | Residues | Atoms | | | | | | AltConf | Trace |
|---|---|---|---|---|---|---|---|---|---|---|
| | | | Total | C | H | N | O | S | | |
| 1 | C | 142 | 2249 | 735 | 1116 | 188 | 207 | 3 | 0 | 0 |
| 1 | D | 143 | 2268 | 741 | 1127 | 189 | 208 | 3 | 0 | 0 |
| 1 | B | 143 | 2268 | 741 | 1127 | 189 | 208 | 3 | 0 | 0 |
| 1 | A | 142 | 2249 | 735 | 1116 | 188 | 207 | 3 | 0 | 0 |

There are 12 discrepancies between the modelled and reference sequences:

| Chain | Residue | Modelled | Actual | Comment | Reference |
|---|---|---|---|---|---|
| C | 48 | ALA | CYS | conflict | UNP A0A0D4D613 |
| C | 61 | ALA | CYS | conflict | UNP A0A0D4D613 |
| C | 107 | ALA | CYS | conflict | UNP A0A0D4D613 |
| D | 48 | ALA | CYS | conflict | UNP A0A0D4D613 |
| D | 61 | ALA | CYS | conflict | UNP A0A0D4D613 |
| D | 107 | ALA | CYS | conflict | UNP A0A0D4D613 |
| B | 48 | ALA | CYS | conflict | UNP A0A0D4D613 |
| B | 61 | ALA | CYS | conflict | UNP A0A0D4D613 |
| B | 107 | ALA | CYS | conflict | UNP A0A0D4D613 |
| A | 48 | ALA | CYS | conflict | UNP A0A0D4D613 |
| A | 61 | ALA | CYS | conflict | UNP A0A0D4D613 |
| A | 107 | ALA | CYS | conflict | UNP A0A0D4D613 |

# 3 Residue-property plots (i)

These plots are drawn for all protein, RNA and DNA chains in the entry. The first graphic for a chain summarises the proportions of the various outlier classes displayed in the second graphic. The second graphic shows the sequence view annotated by issues in geometry. Residues are color-coded according to the number of geometric quality criteria for which they contain at least one outlier: green = 0, yellow = 1, orange = 2 and red = 3 or more. Stretches of 2 or more consecutive residues without any outlier are shown as a green connector. Residues present in the sample, but not in the model, are shown in grey.

• Molecule 1: Core protein

Chain C:

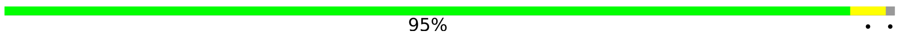


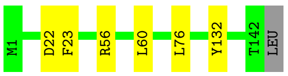


• Molecule 1: Core protein

Chain D:

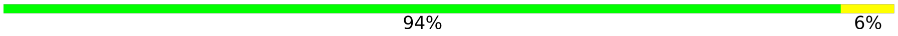


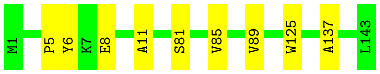


• Molecule 1: Core protein

Chain B:

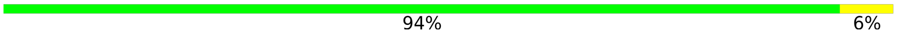


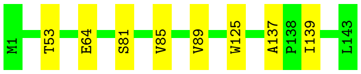


• Molecule 1: Core protein

Chain A:

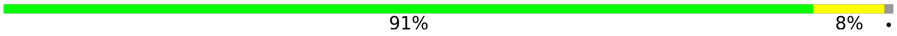


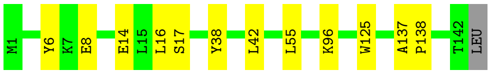

# 4 Experimental information (i)

| Property | Value | Source |
|---|---|---|
| Reconstruction method | SINGLE PARTICLE | Depositor |
| Imposed symmetry | POINT, Not provided | Depositor |
| Number of particles used | 14000 | Depositor |
| Resolution determination method | FSC 0.143 CUT-OFF | Depositor |
| CTF correction method | PHASE FLIPPING AND AMPLITUDE CORRECTION | Depositor |
| Microscope | FEI POLARA 300 | Depositor |
| Voltage (kV) | 300 | Depositor |
| Electron dose $(e^-/\text{Å}^2)$ | 25 | Depositor |
| Minimum defocus (nm) | Not provided | Depositor |
| Maximum defocus (nm) | Not provided | Depositor |
| Magnification | Not provided | Depositor |
| Image detector | GATAN K2 SUMMIT (4k x 4k) | Depositor |

# 5 Model quality (i)

## 5.1 Standard geometry (i)

The Z score for a bond length (or angle) is the number of standard deviations the observed value is removed from the expected value. A bond length (or angle) with $|Z| > 5$ is considered an outlier worth inspection. RMSZ is the root-mean-square of all Z scores of the bond lengths (or angles).

| Mol | Chain | Bond lengths | | Bond angles | |
|---|---|---|---|---|---|
| | | RMSZ | #$\|Z\|$ >2 | RMSZ | #$\|Z\|$ >2 |
| 1 | A | 0.34 | 0/1167 | 0.52 | 0/1599 |
| 1 | B | 0.32 | 0/1175 | 0.56 | 0/1610 |
| 1 | C | 0.33 | 0/1167 | 0.52 | 0/1599 |
| 1 | D | 0.33 | 0/1175 | 0.54 | 0/1610 |
| All | All | 0.33 | 0/4684 | 0.53 | 0/6418 |

There are no bond length outliers.

There are no bond angle outliers.

There are no chirality outliers.

There are no planarity outliers.

## 5.2 Too-close contacts (i)

In the following table, the Non-H and H(model) columns list the number of non-hydrogen atoms and hydrogen atoms in the chain respectively. The H(added) column lists the number of hydrogen atoms added and optimized by MolProbity. The Clashes column lists the number of clashes within the asymmetric unit, whereas Symm-Clashes lists symmetry related clashes.

| Mol | Chain | Non-H | H(model) | H(added) | Clashes | Symm-Clashes |
|---|---|---|---|---|---|---|
| 1 | A | 1133 | 1116 | 1116 | 9 | 0 |
| 1 | B | 1141 | 1127 | 1127 | 7 | 0 |
| 1 | C | 1133 | 1116 | 1116 | 5 | 0 |
| 1 | D | 1141 | 1127 | 1127 | 6 | 0 |
| All | All | 4548 | 4486 | 4486 | 21 | 0 |

The all-atom clashscore is defined as the number of clashes found per 1000 atoms (including hydrogen atoms). The all-atom clashscore for this structure is 2.

All (21) close contacts within the same asymmetric unit are listed below, sorted by their clash magnitude.

| Atom-1 | Atom-2 | Interatomic distance (Å) | Clash overlap (Å) |
|---|---|---|---|
| 1:A:14:GLU:O | 1:A:17:SER:OG | 2.09 | 0.65 |
| 1:D:125:TRP:CZ2 | 1:D:137:ALA:HB2 | 2.42 | 0.55 |
| 1:B:64:GLU:OE2 | 1:A:96:LYS:NZ | 2.39 | 0.55 |
| 1:C:56:ARG:NH2 | 1:D:8:GLU:OE1 | 2.44 | 0.51 |
| 1:A:38:TYR:O | 1:A:42:LEU:HD23 | 2.10 | 0.50 |
| 1:A:125:TRP:CZ2 | 1:A:137:ALA:HB2 | 2.49 | 0.48 |
| 1:C:76:LEU:HD22 | 1:D:81:SER:HB2 | 1.95 | 0.48 |
| 1:D:85:VAL:O | 1:D:89:VAL:HG23 | 2.13 | 0.47 |
| 1:A:42:LEU:HD21 | 1:A:55:LEU:HD23 | 1.99 | 0.45 |
| 1:B:125:TRP:CH2 | 1:B:137:ALA:HB2 | 2.51 | 0.45 |
| 1:B:125:TRP:CZ2 | 1:B:137:ALA:HB2 | 2.51 | 0.45 |
| 1:B:85:VAL:O | 1:B:89:VAL:HG23 | 2.16 | 0.45 |
| 1:C:22:ASP:OD1 | 1:C:23:PHE:N | 2.52 | 0.43 |
| 1:B:81:SER:O | 1:B:85:VAL:HG23 | 2.19 | 0.42 |
| 1:A:125:TRP:CH2 | 1:A:137:ALA:HB2 | 2.55 | 0.42 |
| 1:A:6:TYR:HE2 | 1:A:16:LEU:HD22 | 1.85 | 0.41 |
| 1:C:60:LEU:HD11 | 1:D:5:PRO:HB3 | 2.02 | 0.41 |
| 1:A:137:ALA:HB1 | 1:A:138:PRO:HD2 | 2.03 | 0.41 |
| 1:B:53:THR:HG23 | 1:A:8:GLU:OE2 | 2.21 | 0.41 |
| 1:D:6:TYR:HB3 | 1:D:11:ALA:HB3 | 2.03 | 0.40 |
| 1:C:132:TYR:O | 1:B:139:ILE:HG22 | 2.22 | 0.40 |

There are no symmetry-related clashes.

## 5.3 Torsion angles (i)

### 5.3.1 Protein backbone (i)

In the following table, the Percentiles column shows the percent Ramachandran outliers of the chain as a percentile score with respect to all PDB entries followed by that with respect to all EM entries.

The Analysed column shows the number of residues for which the backbone conformation was analysed, and the total number of residues.

| Mol | Chain | Analysed | Favoured | Allowed | Outliers | Percentiles | |
|---|---|---|---|---|---|---|---|
| 1 | A | 140/143 (98%) | 134 (96%) | 6 (4%) | 0 | 100 | 100 |
| 1 | B | 141/143 (99%) | 132 (94%) | 9 (6%) | 0 | 100 | 100 |
| 1 | C | 140/143 (98%) | 135 (96%) | 5 (4%) | 0 | 100 | 100 |
| 1 | D | 141/143 (99%) | 131 (93%) | 10 (7%) | 0 | 100 | 100 |
| All | All | 562/572 (98%) | 532 (95%) | 30 (5%) | 0 | 100 | 100 |

There are no Ramachandran outliers to report.

### 5.3.2 Protein sidechains (i)

In the following table, the Percentiles column shows the percent sidechain outliers of the chain as a percentile score with respect to all PDB entries followed by that with respect to all EM entries.

The Analysed column shows the number of residues for which the sidechain conformation was analysed, and the total number of residues.

| Mol | Chain | Analysed | Rotameric | Outliers | Percentiles | |
|---|---|---|---|---|---|---|
| 1 | A | 123/124 (99%) | 123 (100%) | 0 | 100 | 100 |
| 1 | B | 124/124 (100%) | 124 (100%) | 0 | 100 | 100 |
| 1 | C | 123/124 (99%) | 123 (100%) | 0 | 100 | 100 |
| 1 | D | 124/124 (100%) | 124 (100%) | 0 | 100 | 100 |
| All | All | 494/496 (100%) | 494 (100%) | 0 | 100 | 100 |

There are no protein residues with a non-rotameric sidechain to report.

Some sidechains can be flipped to improve hydrogen bonding and reduce clashes. There are no such sidechains identified.

### 5.3.3 RNA (i)

There are no RNA molecules in this entry.

## 5.4 Non-standard residues in protein, DNA, RNA chains (i)

There are no non-standard protein/DNA/RNA residues in this entry.

## 5.5 Carbohydrates (i)

There are no carbohydrates in this entry.

## 5.6 Ligand geometry (i)

There are no ligands in this entry.

## 5.7 Other polymers (i)

There are no such residues in this entry.

## 5.8 Polymer linkage issues (i)

There are no chain breaks in this entry.
